# New Casbane Diterpenoids from a South China Sea Soft Coral, *Sinularia* sp

**DOI:** 10.3390/md11020455

**Published:** 2013-02-06

**Authors:** Jian Yin, Min Zhao, Minshan Ma, Yuping Xu, Zheng Xiang, Yuepiao Cai, Jianyong Dong, Xinxiang Lei, Kexin Huang, Pengcheng Yan

**Affiliations:** 1 School of Pharmacy, Wenzhou Medical College, Wenzhou 325035, China; E-Mails: yinjian198708@gmail.com (J.Y.); miniezhao@gmail.com (M.Z.); zjxuyuping@gmail.com (Y.X.); xzh007@126.com (Z.X.); ypcai@wzmc.edu.cn (Y.C.); jianyd@wzmc.edu.cn (J.D.); 2 Analytical and Testing Center, Wenzhou University, Wenzhou 325035, China; E-Mails: youihj1023@gmail.com (M.M.); xxlei@wzu.edu.cn (X.L.)

**Keywords:** soft coral, *Sinularia*, casbane diterpenoids, cytotoxicity, NO inhibition

## Abstract

Six new casbane diterpenoids, named as sinularcasbanes A–F (**1**–**6**), along with six known analogues **7**–**12**, were isolated from a South China Sea soft coral, *Sinularia* sp. The structures of the new compounds were elucidated by extensive spectroscopic analysis and by comparison with data reported in the literature. All compounds were evaluated for their cytotoxicity against selected cancer cell lines and the inhibition of lipopolysaccharide (LPS)-induced nitric oxide (NO) production in mouse peritoneal macrophages.

## 1. Introduction

The casbanes are a small group of diterpenoids strictly related to the cembrane skeleton, and characterized by the presence of a dimethyl-cyclopropyl moiety fused to the 14-membered ring. To date, approximately 33 casbane diterpenoids, in the majority of which the two rings forming the macrocyclic structure are *cis*-fused, have been isolated and described mainly from some plants of the family Euphorbiaceae [1,2,3,4,5,6,7,8,9,10,11,12,13], as well as from two species of soft coral belonging to the genus *Sinularia* (*S. microclavata* and *S. depressa*) [14,15]. The biological potential of casbane diterpenoids has not been explored extensively, however some members of this group have been proven to display moderate cytotoxicity and antimicrobial activity [14,15].

**Figure 1:**
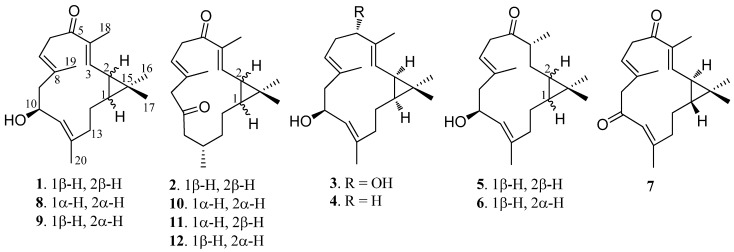
Structures of compounds **1**–**12**.

Soft corals belonging to the genus *Sinularia* distribute widely in the tropical reef, and have been shown to be a plentiful source of diterpenoids, possessing a range of biological activities [16,17]. Our recent study of bioactive natural products from a Hainan soft coral, *Sinularia* sp., resulted in the isolation of six new casbane diterpenoids **1**–**6**, along with six known analogues **7**–**12** (Figure 1). The compounds isolated were evaluated for their cytotoxicity against selected cancer cell lines and their inhibitory activity against lipopolysaccharide (LPS)-induced nitric oxide (NO) production in mouse peritoneal macrophages (PEM*Φ*). Herein we report the purification, structural elucidation, and bioactivity of these compounds.

## 2. Results and Discussion

Sinularcasbane A (**1**) had a molecular formula of C_2__0_H_30_O_2_ as determined by HRESIMS *m*/*z*325.2142 [M + Na]^+^ (Calcd. 325.2138) and NMR data, requiring six degrees of unsaturation. The ^1^H NMR spectrum of **1** showed resonances for five methyl singlets including three olefinic methyls [*δ*_H_ 1.58 (3H, s), 1.68 (3H, s), and 1.88 (3H, s)] and two tertiary methyls [*δ*_H_ 1.06 (3H, s), 1.17 (3H, s)], while the ^13^C NMR spectrum displayed 20 carbon resonances including a carbonyl and six olefinic carbons (Table 1, Table 2). The IR absorption at 1655 cm^−1^ and ^13^C NMR signal at *δ*_C_ 199.8 (C-5) indicated the presence of an α,β-unsaturated ketone group. Six olefinic carbon signals in the ^13^C NMR spectrum at *δ*_C_ 142.6 (CH-3), 139.8 (C-12), 137.2 (C-4), 134.1 (C-8), 127.1 (CH-11), and 122.0 (CH-7), and three olefinic proton signals in the ^1^H NMR spectrum at *δ*_H_ 6.30 (1H, d, *J* = 10.0 Hz, H-3), 5.09 (1H, br d, *J* = 9.0 Hz, H-7), and 4.98 (1H, d, *J* = 9.5 Hz, H-11) were attributed to three trisubstituted double bonds. In addition, a secondary hydroxyl group was observed from an oxymethine proton signal at *δ*_H_ 4.53 (1H, dt, *J* = 5.0, 9.5 Hz, H-10) and carbon resonance at *δ*_C_ 66.2 (CH-10). Four degrees of unsaturation, accounted for by the functional groups from six in the molecule, suggested a bicyclic structure in **1**. Moreover, a dimethyl-cyclopropyl moiety was established by HMBC correlations from the two tertiary methyls mentioned above to a quaternary carbon (*δ*_C_ 25.8, C-15) and two methine carbons [*δ*_C_ 34.6 (CH-1), 27.3 (CH-2)], in association with COSY correlations between two methine protons [*δ*_H_ 1.14 (1H, m, H-1), 1.51 (1H, t, *J* = 9.0 Hz, H-2)], suggesting a casbane skeleton for **1**. Furthermore, detailed 2D NMR data analysis revealed that **1** shared the same gross structure as 10-hydroxydepressin (**8**) [15]. The difference found in the significantly upfield shifted C-20 (*δ*_C_15.0) compared to **8** (*δ*_C_ 18.3), implying compound **1** was a diastereomer of **8**. The *E* geometry of all three double bonds of **1** was inferred by the chemical shifts of C-18, C-19, and C-20 (<20 ppm) [15]. The junction of the cyclopropyl ring at carbons C-1/C-2 was suggested to be *cis* based from the diagnostic ^13^C NMR chemical shifts of C-16 and C-17 (*δ*_C_ 15.9 and 29.0, respectively), in accordance with those reported for **8**. On biogenetic considerations, the same relative configuration of C-10 was assumed for **1**. Consequently, the relative configurations of C-1 and C-2 in **1** had to be opposite to those of **8** for the diastereomeric relationship. Due to the reversed ring junction, **1** was found to have a rotation of positive phase ([α]^25^_D_ +127.1) in contrast to that of **8**.

**Table 1:** ^13^C NMR data for compounds **1**–**6** (CDCl_3_, 125 MHz, *δ*_C_).

No.	1	2	3	4	5	6
1	34.6 (CH)	34.8 (CH)	31.5 (CH)	30.7 (CH)	24.4 (CH)	28.1 (CH)
2	27.3 (CH)	27.6 (CH)	25.7 (CH)	25.9 (CH)	24.6 (CH)	24.8 (CH)
3	142.6 (CH)	144.0 (CH)	125.5 (CH)	121.2 (CH)	28.5 (CH_2_)	30.2 (CH_2_)
4	137.2 (C)	135.8 (C)	136.9 (C)	135.8 (C)	43.9 (CH)	46.5 (CH)
5	199.8 (C)	199.5 (C)	79.3 (CH)	39.4 (CH_2_)	214.8 (C)	211.9 (C)
6	39.1 (CH_2_)	40.3 (CH_2_)	33.2 (CH_2_)	25.0 (CH_2_)	42.8 (CH_2_)	40.5 (CH_2_)
7	122.0 (CH)	123.6 (CH)	121.9 (CH)	127.0 (CH)	120.3 (CH)	120.0 (CH)
8	134.1 (C)	129.9 (C)	132.5 (C)	130.8 (C)	136.6 (C)	134.5 (C)
9	47.5 (CH_2_)	52.2 (CH_2_)	47.8 (CH_2_)	48.0 (CH_2_)	47.5 (CH_2_)	47.2 (CH_2_)
10	66.2 (CH)	206.8 (C)	66.5 (CH)	66.9 (CH)	66.5 (CH)	67.4 (CH)
11	127.1 (CH)	47.4 (CH_2_)	127.7 (CH)	127.0 (CH)	126.8 (CH)	128.5 (CH)
12	139.8 (C)	26.7 (CH)	140.1 (C)	140.1 (C)	140.2 (C)	138.6 (C)
13	39.9 (CH_2_)	33.9 (CH_2_)	40.0 (CH_2_)	39.5 (CH_2_)	38.0 (CH_2_)	40.6 (CH_2_)
14	25.4 (CH_2_)	19.7 (CH_2_)	24.3 (CH_2_)	23.9 (CH_2_)	21.1 (CH_2_)	24.9 (CH_2_)
15	25.8 (C)	25.1 (C)	20.7 (C)	20.0 (C)	17.1 (C)	19.6 (C)
16	15.9 (CH_3_)	16.0 (CH_3_)	28.8 (CH_3_)	28.9 (CH_3_)	14.9 (CH_3_)	21.5 (CH_3_)
17	29.0 (CH_3_)	29.1 (CH_3_)	15.5 (CH_3_)	15.6 (CH_3_)	29.2 (CH_3_)	22.1 (CH_3_)
18	11.7 (CH_3_)	11.3 (CH_3_)	10.2 (CH_3_)	16.0 (CH_3_)	17.3 (CH_3_)	13.4 (CH_3_)
19	16.6 (CH_3_)	18.6 (CH_3_)	17.2 (CH_3_)	17.1 (CH_3_)	16.9 (CH_3_)	17.8 (CH_3_)
20	15.0 (CH_3_)	20.2 (CH_3_)	18.5 (CH_3_)	18.6 (CH_3_)	17.2 (CH_3_)	16.4 (CH_3_)

**Table 2:** ^1^H NMR data for compounds **1**–**6** (CDCl_3_, 500 MHz, *δ*_H_, *J* in Hz).

No.	1	2	3	4	5	6
1	1.14 m	1.02 m	0.73 m	0.68 m	0.19 m	0.06 m
2	1.51 t (9.0)	1.46 dd (8.0, 10.0)	1.27 t (9.0)	1.24 t (8.5)	0.17 m	0.16 m
3	6.30 d (10.0)	6.28 d (10.0)	5.06 d (9.5)	4.86 d (9.0)	0.95 m 1.80 m	1.60 m 1.98 m
4	-	-	-	-	3.05 m	2.70 m
5	-	-	4.03 dd (4.0, 11.0)	2.09 m	-	-
				2.21 m		
6	2.90 br d (14.0)	3.00 br d (15.0)	2.27 m	2.07 m	3.03 dd (7.5, 15.5)	2.76 dd (6.5, 14.5)
	3.62 dd (9.0, 14.0)	3.66 dd (8.0, 15.0)	2.38 m	2.20 m	3.19 dd (7.5, 15.5)	3.34 dd (8.5, 14.5)
7	5.09 br d (9.0)	5.29 br d (8.0)	4.74 t (6.5)	4.89 t (6.0)	5.32 t (7.5)	5.15 t (7.0)
9	2.12 m	2.93 d (18.0)	2.10 dd (11.0, 13.5)	2.10 m	2.36 dd (7.5, 13.0)	2.10 m
	2.46 br d (12.0)	2.95 d (18.0)	2.35 m	2.37 m	2.47 dd (4.0, 13.0)	2.50 br d (14.0)
10	4.53 dt (5.0, 9.5)	-	4.52 dt (2.5, 9.0)	4.55 dt (3.5, 9.0)	4.66 dt (4.0, 8.5)	4.69 dt (3.5, 9.0)
11	4.98 d (9.5)	2.17 dd (3.0, 18.0)	4.98 d (9.0)	5.00 d (9.0)	5.37 d (8.5)	4.93 d (9.0)
		2.59 dd (10.0, 18.0)				
12	-	2.33 m	-	-	-	-
13	1.80 m	1.28 m	1.85 m	1.83 m	2.08 m	1.79 m
	2.23 m	1.40 m	2.29 m	2.27 m	2.12 m	2.11 m
14	0.77 m	0.97 m	1.05 m	1.15 m	1.23 m	1.03 m
	2.10 m	1.43 m	1.74 m	1.73 m	1.72 m	1.48 m
16	1.06 s	1.10 s	1.08 s	1.09 s	0.87 s	0.97 s
17	1.17 s	1.15 s	0.97 s	0.98 s	0.96 s	1.02 s
18	1.88 s	1.81 s	1.65 s	1.64 s	1.07 d (7.0)	1.04 d (7.0)
19	1.58 s	1.82 s	1.63 s	1.61 s	1.69 s	1.74 s
20	1.68 s	0.96 d (7.0)	1.68 s	1.68 s	1.78 s	1.73 s

The HRESIMS of sinularcasbane B (**2**) exhibited a pseudomolecular ion peak at *m*/*z* 325.2134 [M + Na]^+^, consistent with the molecular formula of C_20_H_30_O_2_. Analysis of the ^1^H and ^13^C NMR spectra of **2** clearly revealed the presence of two trisubstituted double bonds [*δ*_H_ 6.28 (1H, d, *J* = 10.0 Hz, H-3), 5.29 (1H, br d, *J* = 8.0 Hz, H-7); *δ*_C_ 144.0 (CH-3), 135.8 (C-4), 129.9 (C-8), and 123.6 (CH-7)], two ketones [*δ*_C_ 206.8 (C-10), 199.5 (C-5)], and five methyls including two olefinic methyls [*δ*_H_ 1.82 (3H, s, H_3_-19), 1.81 (3H, s, H_3_-18)], two tertiary methyls [*δ*_H_ 1.10 (3H, s, H_3_-16), 1.15 (3H, s, H_3_-17)], and a secondary methyl [*δ*_H_ 0.96 (3H, d, *J* = 7.0 Hz, H_3_-20)]. Further interpretation of 1D and 2D NMR spectroscopic data confirmed that **2** shared the same gross structure as 10-oxo-11,12-dihydrodepressin (**10**), and its two isomers 2-*epi*-10-oxo-11,12-dihydrodepressin (**11**) and 1-*epi*-10-oxo-11,12-dihydrodepressin (**12**) [15]. The *E* geometry of two double bonds Δ^3(4)^ and Δ^7(8)^, and the *cis* junction of the cyclopropyl moiety in **2** was determined on the basis of similar NMR data in comparison with those of **10**. In addition, the ^13^C NMR chemical shifts of C-11 (*δ*_C_ 47.4), C-13 (*δ*_C_ 33.9), C-14 (*δ*_C_ 19.7), and C-20 (*δ*_C_ 20.2) were significantly shifted compared to those reported for **10** (*δ*_C_ 50.1, 39.9, 24.5, and 23.1, respectively), implying **2** had to differ from **10** in the relative configuration of one or two chiral centers. On the other hand, it was found that **2** and **10** had rotations of opposite sign, allowing the assignment of a different ring junction. Thus, H-1 and H-2 in **2** were suggested to be β-oriented.

**Figure 2:**
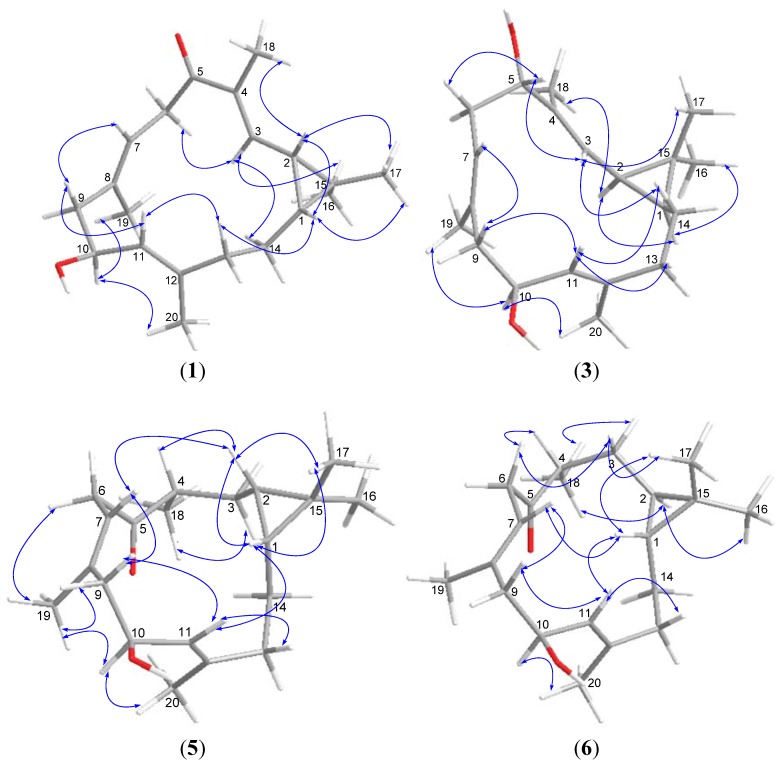
NOESY correlations of compounds **1**, **3**, **5**, and **6**.

Sinularcasbane C (**3**) has a molecular formula of C_20_H_32_O_2_ as determined by HRESIMS data (*m*/*z* 327.2267 [M + Na]^+^), requiring five degrees of unsaturation. The analysis of 1D and 2D NMR spectroscopic data revealed that the gross structure of **3** was closely related to 10-hydroxydepressin (**8**) [15], with the exception that C-5 was linked to a hydroxyl group instead of a ketone in the latter. This was indicated by the presence of an additional oxygenated methine group [*δ*_H_ 4.03 (1H, dd, *J* = 4.0, 11.0 Hz, H-5); *δ*_C_ 79.3 (CH-5)], and its proton exhibiting HMBC correlations to C-18 (*δ*_C_ 10.2, CH_3_), C-3 (*δ*_C_ 125.5, CH), and C-4 (*δ*_C_ 136.9, C), and in turn the correlations of H_3_-18 (*δ*_H_ 1.65, s) to C-5 (*δ*_C_ 79.3, CH), C-3, and C-4. The geometry of three double bonds and stereogenic centers at C-1, C-2, and C-10 was in agreement with those of **8** based on the similar NOE relationships and NMR data in association with the chemical constants. The coupling constant *J*_H-10/H-11_ of **3** was 9.0 Hz, suggesting H-10 (*δ*_H_ 4.52, 1H, dt, *J* = 2.5, 9.0 Hz) and H-11 (*δ*_H_ 4.98, 1H, d, *J* = 9.0 Hz) were oriented in the *trans*-axial configuration. The obvious NOE correlations of H-11/H-14a (*δ*_H_ 1.05, m), H-14a/H-3 (*δ*_H_ 5.06, 1H, d, *J* = 9.0 Hz), and H-3/H-5 led to the assignment of H-5β, while the NOE interactions of H_3_-18/H-2 (*δ*_H_ 1.27, 1H, t, *J* = 9.0 Hz) and H-2/H-1 (*δ*_H_ 0.73, 1H, m) supported the α-orientation of H-1 and H-2 (Figure 2).

The NMR spectroscopic data of sinularcasbane D (**4**) (Table 1, Table 2) indicated that its structure was closely related to that of **3**. The only difference was found to be the dehydroxylation of C-5 in **4** compared to **3**, as evident from the HMBC correlation from H_3_-18 (*δ*_H_ 1.64, s) to a methylene carbon C-5 (*δ*_C_ 39.4), instead of a hydroxy-bearing methine carbon (*δ*_C_ 79.3) as in **3**. The molecular formula of **4**, C_20_H_32_O as established by HRESIMS (*m*/*z* 311.2322 [M + Na]^+^), was 16 amu less than that of **3** and further supported this structure assignment. The relative stereochemistry of **4** was in agreement with that of **3** based on the similar NMR and NOE data. Thus, compound **4** was defined as a C-5 dehydroxylated analogue of **3**.

**Figure 3:**
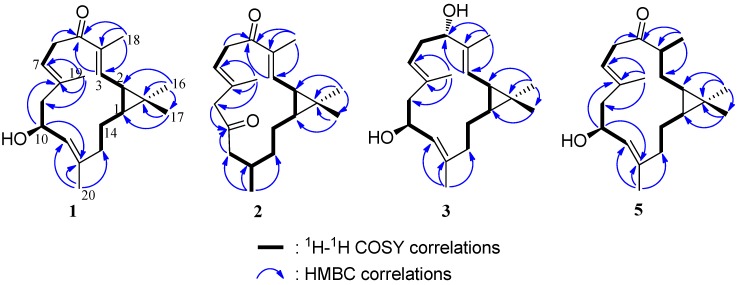
COSY and HMBC correlations of compounds **1**–**3** and **5**.

The molecular formula of sinularcasbane E (**5**) was determined as C_20_H_32_O_2_ based on HRESIMS data (*m*/*z* 327.2286 [M + Na]^+^, Calcd. 327.2295), implying five degrees of unsaturation. The NMR data of **5** were compatible with those of **1**, except for the presence of an additional secondary methyl group [*δ*_H_ 1.07 (3H, d, *J* = 7.0 Hz, H_3_-18); *δ*_C_ 17.3 (CH_3_-18)]. The secondary methyl group was assigned to C-4 as evident from HMBC correlations of its protons H_3_-18 to ketone carbon C-5 (*δ*_C_ 214.8, C), methylene carbon C-3 (*δ*_C_ 28.5, CH_2_), and methine carbon C-4 (*δ*_C_ 43.9, CH), in association with the COSY correlations between H_3_-18/H-4 (*δ*_H_ 3.05, 1H, m), H-4/H_2_-3 (*δ*_H_ 0.95, 1H, m; 1.80, 1H, m), and H_2_-3/H-2 (*δ*_H_ 0.17, 1H, m). In addition, the remaining substructures from C-6 to C-14 together with the cyclopropyl moiety were confirmed to resemble those of **1** by HMBC and COSY correlations as shown in Figure 3. The NOE relationships between H-10 (*δ*_H_ 4.66, dt, *J* = 4.0, 8.5 Hz)/H_3_-20 (*δ*_H_ 1.78, s), and H-10/H_3_-19 (*δ*_H_ 1.69, s) indicated these protons approximated spatially and oriented in the same face, whereas the key NOE interactions between H-7 (*δ*_H_ 5.32, t, *J* = 7.5 Hz)/H-2, H-11 (*δ*_H_ 5.37, d, *J* = 8.5 Hz)/H-1 (*δ*_H_ 0.19, m), H-1/H-2, and H-2/H-4 supported the β-orientation of H-1, H-2 and H-4.

Sinularcasbane F (**6**) had the same molecular formula as that of **5**, as determined by HRESIMS and NMR data (Table 1, Table 2). It was determined to be a stereoisomer of **5** based on the extensive 2D NMR spectroscopic analysis and by comparison of its NMR data with those of **5**. The difference was found in the ^13^C NMR values of C-16 (*δ*_C_ 21.5, CH_3_) and C-17 (*δ*_C_ 22.1, CH_3_) that were significantly shifted in comparison with those of **5**, suggesting **6** had a different ring junction at carbons C-1/C-2. This assignment was further confirmed by the presence of NOE cross-peaks between H-1 (*δ*_H_ 0.06, m) and H_3_-17 (*δ*_H_ 1.02, s), and between H-2 (*δ*_H_ 0.16, m) and H_3_-16 (*δ*_H_ 0.97, s), consistent with a *trans* ring junction. In addition, the NOE correlations of H-10 (*δ*_H_ 4.69, dt, *J* = 3.5, 10.0 Hz)/H_3_-20 (*δ*_H_ 1.73, s) and H-11 (*δ*_H_ 4.93, d, *J* = 9.0 Hz)/H-1 indicated the β-orientation of H-1, while the NOE relationship between H-2 and H_3_-18 (*δ*_H_ 1.04, d, *J* = 7.0 Hz) suggested both H-2 and H_3_-18 to be α-oriented.

The identities of compounds **7**–**12** were established by comparison of their spectral data with those of the known compounds isolated from *S. depressa* [15]. They are 1-*epi*-10-oxodepressin (**7**), 10-hydroxydepressin (**8**), 1-*epi*-10-hydroxydepressin (**9**), 10-oxo-11,12-dihydrodepressin (**10**), 2-*epi*-10-oxo-11,12-dihydrodepressin (**11**), and 1-*epi*-10-oxo-11,12-dihydrodepressin (**12**).

Compounds **1**–**12** were tested for their cytotoxicity against a panel of tumor cell lines including SGC7901 (human gastric carcinoma), A549 (human lung epithelial carcinoma), MCF7 (human breast carcinoma), HCT116 (human colonic carcinoma), B16 (mouse melanoma) and P815 (mouse lymphoblast-like mastocytoma). However, they showed no cytotoxic activity against all cell lines at 50 μM. In order to detect the anti-inflammatory property of these compounds, the test for inhibition of lipopolysaccharide (LPS)-induced nitric oxide (NO) production in mouse peritoneal macrophages (PEM*Φ*) was performed. The bioassay results revealed compounds **2** and **5** showed moderate inhibition with an inhibitory concentration 50% (IC_50_) of 8.3 and 5.4 μM, respectively, whereas the other compounds showed weak activity (IC_50_ > 10 μM). In addition, all compounds were weakly cytotoxic toward mouse PEM*Φ* (IC_50_ > 10 μM).

## 3. Experimental Section

### 3.1. General Experimental Procedures

Optical rotations were measured on a PoLAAR 3005 digital polarimeter. IR spectra were determined on a Bruker Equinox 55 spectrometer. ^1^H and ^13^C NMR and 2D NMR were recorded on a Bruker Avance 500 MHz NMR spectrometer using TMS as an internal standard. *δ* values were expressed in parts per million (ppm), and *J* values were reported in Hertz (Hz). HRESIMS data were obtained from a Bruker micrO TOF-QII instrument. Silica gel (200–300 mesh) for column chromatography and GF_254_ silica gel for TLC was provided by Qingdao Marine Chemistry Co. Ltd. Reversed-phase HPLC chromatography was performed on an Agilent 1100 series instrument using a VWD G1314A detector at 210 nm and a YMC-Pack C_18_ (10 μm, 250 × 10 mm) column.

### 3.2. Animal Material

The soft coral *Sinularia* sp. was collected off the coast of Ximao island, Hainan Province, China, in November 2011, at a depth of 8 m and was frozen immediately after collection. The specimen was identified by Xiu-Bao Li (South China Sea Institute of Oceanology, CAS, Guangzhou, China). A voucher specimen (HS201101) is deposited at the Institute of Natural Drugs Development, Wenzhou Medical College, China.

### 3.3. Extraction and Isolation

The frozen soft coral *Sinularia* sp. (2.7 kg) was homogenized and then extracted with 95% EtOH (4 × 3 L) at room temperature. The EtOH extract (171.5 g) was partitioned between H_2_O and EtOAc. The EtOAc fraction (40.0 g) was subjected to silica gel (200–300 mesh) column chromatography, and was eluted with a gradient of petroleum ether (PE)/EtOAc (20:1, 10:1, 5:1, 1:1) to obtain nine fractions (F1–F9). F5 (2.7 g) was fractioned on Sephadex LH-20 (70 × 2.5 cm, eluted with CH_2_Cl_2_/MeOH 1:1), and further fractioned on an ODS column (C_18_, 25 × 2 cm), eluted with a gradient of MeOH/H_2_O (70:30–100:0) to afford seven subfractions (F4A–F4G). The subfraction F4A (62 mg) was further separated on reversed-phase semi-preparative HPLC with MeOH/H_2_O (75:25) as a mobile phase to obtain **11** (10.0 mg). F4D (108 mg) was purified by HPLC (MeOH/H_2_O, 80:20) to afford **7** (3.7 mg), **2** (6.8 mg), **12** (10.7 mg), and **10** (7.9 mg). F4G (53 mg) was purified by HPLC (MeOH/H_2_O, 90:10) to afford **4** (9.0 mg). F6 (0.7 g) was subjected to Sephadex LH-20 column eluting with CH_2_Cl_2_/MeOH (1:1) to afford six subfractions (F6A–F6F). The subfraction F6D (99 mg) was purified by HPLC (MeOH/H_2_O, 80:20) to afford **5** (14.8 mg) and **6** (19.0 mg). F7 (1.6 g) was subjected to Sephadex LH 20 (70 × 2.5 cm, eluted with CH_2_Cl_2_/MeOH 1:1), and further separated on an ODS column (C_18_, 25 × 2 cm), eluted with a gradient of MeOH/H_2_O (75:25–100:0) to furnish five subfractions (F7A–F7E). The subfraction F7C (29 mg) was purified by HPLC (MeOH/H_2_O, 80:20) to afford **3** (10.0 mg), while compounds **1** (2.1 mg), **8** (25.2 mg), and **9** (46.3 mg) were obtained from F7D (114 mg) by the same separation process as that for F7C.

Sinularcasbane A (**1**), obtained as colorless oil; [α]^25^_D_ +127.1 (*c* 0.21, CHCl_3_); IR (KBr) ν_max_3434, 2926, 2859, 1655, 1448, 1384, 1274, 1070, 1017 cm^−1^; ^1^H and ^13^C NMR data, see Table 1, Table 2; HRESIMS (*m*/*z*) 325.2142 [M + Na]^+^ (Calcd. for C_2__0_H_30_O_2_Na, 325.2138).

Sinularcasbane B (**2**), obtained as colorless oil; [α]^25^_D_ −29.0 (*c* 0.60, CHCl_3_); IR (KBr) ν_max_ 2927, 2863, 1708, 1627, 1457, 1376, 1278, 1112, 1061 cm^−1^; ^1^H and ^13^C NMR data, see Table 1, Table 2; HRESIMS (*m*/*z*) 325.2134 [M + Na]^+^ (Calcd. for C_2__0_H_30_O_2_Na, 325.2138).

Sinularcasbane C (**3**), obtained as colorless oil; [α]^25^_D_ −247.6 (*c* 0.24, CHCl_3_); IR (KBr) ν_max_3262, 2923, 2856, 1445, 1384, 1003 cm^−1^; ^1^H and ^13^C NMR data, see Table 1, Table 2; HRESIMS (*m*/*z*) 327.2267 [M + Na]^+^ (Calcd. for C_2__0_H_32_O_2_Na, 327.2295).

Sinularcasbane D (**4**), obtained as colorless oil; [α]^25^_D_ −127.6 (*c* 0.20, CHCl_3_); IR (KBr) ν_max_3435, 2927, 2860, 1451, 1378, 1378 cm^−1^; ^1^H and ^13^C NMR data, see Table 1, Table 2; HRESIMS (*m*/*z*) 311.2322 [M + Na]^+^ (Calcd. for C_2__0_H_32_ONa, 311.2345).

Sinularcasbane E (**5**), obtained as colorless oil; [α]^25^_D_ +171.8 (*c* 0.30, CHCl_3_); IR (KBr) ν_max_3434, 2927, 2860, 1704, 1453, 1379, 1014 cm^−1^; ^1^H and ^13^C NMR data, see Table 1, Table 2; HRESIMS (*m*/*z*) 327.2286 [M + Na]^+^ (Calcd. for C_2__0_H_32_O_2_Na, 327.2295).

Sinularcasbane F (**6**), obtained as colorless oil; [α]^25^_D_ −166.7(*c* 0.35, CHCl_3_); IR (KBr) ν_max_3445, 2927, 2860, 1707, 1626, 1455, 1379, 1043 cm^−1^; ^1^H and ^13^C NMR data, see Table 1, Table 2; HRESIMS (*m*/*z*) 327.2288 [M + Na]^+^ (Calcd. for C_2__0_H_32_O_2_Na, 327.2295).

### 3.4. Cytotoxicity Assay

The cytotoxicity properties of the isolated compounds were tested *in vitro* using human tumor cell lines including SGC7901 (human gastric carcinoma), A549 (human lung epithelial carcinoma), MCF7 (human breast carcinoma), HCT116 (human colonic carcinoma), B16 (mouse melanoma) and P815 (mouse lymphoblast-like mastocytoma) tumor cells by a modification of the MTT colorimetric method according to a previously described procedure [18,19]. The cell lines were purchased from the Cell Resource Center of Shanghai Institute of Biological Sciences, CAS.

### 3.5. Assay for Inhibition of Nitric Oxide (NO) Production and Cytotoxicity against Mouse Peritoneal Macrophages (PEM*Φ*)

Dexamethasone (DEX, positive control, 20 mM in DMSO) and each compound (20 mM in DMSO) were diluted to 1–20 μM range at r.t. before the experiment. The final percentage of DMSO in the reaction mixture was less than 0.5% (v/v). LPS (1 μg/mL), 4% sodium thioglycollate, RPMI1640, fetal bovine serum (FBS), phosphate buffered saline (PBS), MTT and Griess reagents were purchased from Sigma (St, Louis, MO, USA). Mouse peritoneal macrophages (PEM*Φ*) were obtained from C57BL6J male mice, and then plated onto 48 well plates and cultured for 2 h in Dulbecco’s modified Eagle’s medium (DMEM) containing 5% FBS at 5% CO_2_ at 37 °C. Mouse PEM*Φ* were incubated with the test compounds for 1 h at 37 °C before stimulation with 1 μg/mL of lipopolysaccharide (LPS) for 24 h. In the primary test, blank control (enchylema) and LPS were added with the compound (1 μM), and DEX (1 μM) was prepared. Cells (5 × 10^5^ cells) were preincubated at 37 °C for 24 h in serum-free medium, and NO production was monitored by measuring nitrite levels in culture media using Griess reagent. Absorbance was measured at 548 nm in incubated media with Griess reagent for 10 min. Viable adherent cells were stained with MTT (2 μg/mL) for 4 h. Media was then removed and the formazan crystals produced were dissolved in DMSO (200 μL). Absorbance was tested at 540 nm. The cytotoxicity against PEM*Φ* was tested by MTT colorimetry [18].

One-way analysis of variance was applied for all statistical analysis by independent experiments, and data were represented as means ± standard error of the measurement. Individual values were compared by *t*-test and a *p*-value <0.01 to evaluate the significance.

## 4. Conclusions

Sinularcasbanes A–F (**1**–**6**) together with six known casbane diterpenoids **7**–**12** were isolated from the EtOAc extract of the South China Sea soft coral *Sinularia* sp. All of the compounds exhibited no cytotoxic activity against SGC7901, A549, MCF7, HCT116, B16, and P815 tumor cells at 50 μM in the *in vitro* cytotoxicity assay. Sinularcasbanes B (**2**) and E (**5**) were found to show moderate inhibitory activity against lipopolysaccharide (LPS)-induced nitric oxide (NO) production in mouse peritoneal macrophages (PEM*Φ*) with an IC_50_ of 8.3 and 5.4 μM, respectively. NO plays an important role in the inflammatory process, therefore, sinularcasbanes B (**2**) and E (**5**) could be promising lead compounds for anti-inflammatory agents. Other studies should be performed to elucidate the mechanism by which these compounds inhibit the production of NO. A literature survey revealed that sinularcasbanes E (**5**) and F (**6**) are the first members of the casbane family with a carbon-carbon single bond between carbons C3 and C4. The present work provided a group of new casbane diterpenoids with which to enrich the chemical diversity of *Sinularia* corals.

## Supplementary Files

Supplementary File 1 Supplementary Information (PDF, 1305 KB)
