# Supplementary material for: New Casbane Diterpenoids from a South China Sea Soft Coral, Sinularia sp
Source: Mar Drugs. 2013 Feb 6;11(2):455–65. doi: 10.3390/md11020455 (PMC3640392; doi:10.3390/md11020455)

Supplementary Information

Figure S1. ^{1}H NMR spectrum (500 MHz) of compound **1** in $CDCl_3$. 3
Figure S2. ^{13}C NMR spectrum (125 MHz) of compound **1** in $CDCl_3$. 3
Figure S3. HSQC spectrum (500 MHz) of compound **1** in $CDCl_3$. 4
Figure S4. HMBC spectrum (500 MHz) of compound **1** in $CDCl_3$. 4
Figure S5. COSY spectrum (500 MHz) of compound **1** in $CDCl_3$. 5
Figure S6. NOESY spectrum (500 MHz) of compound **1** in $CDCl_3$. 5
Figure S7. IR spectrum of compound **1**. 6
Figure S8. HR-ESIMS spectrum of compound **1**. 6
Figure S9. ^{1}H NMR spectrum (500 MHz) of compound **2** in $CDCl_3$. 7
Figure S10. ^{13}C NMR spectrum (125 MHz) of compound **2** in $CDCl_3$. 7
Figure S11. HSQC spectrum (500 MHz) of compound **2** in $CDCl_3$. 8
Figure S12. HMBC spectrum (500 MHz) of compound **2** (**2**) in $CDCl_3$. 8
Figure S13. COSY spectrum (500 MHz) of compound **2** in $CDCl_3$. 9
Figure S14. NOESY spectrum (500 MHz) of compound **2** in $CDCl_3$. 9
Figure S15. IR spectrum of compound **2**. 10
Figure S16. HR-ESIMS spectrum of compound **2**. 10
Figure S17. ^{1}H NMR spectrum (500 MHz) of compound **3** in $CDCl_3$. 11
Figure S18. ^{13}C NMR spectrum (125 MHz) of compound **3** in $CDCl_3$. 11
Figure S19. HSQC spectrum (500 MHz) of compound **3** in $CDCl_3$. 12
Figure S20. HMBC spectrum (500 MHz) of compound **3** in $CDCl_3$. 12
Figure S21. COSY spectrum (500 MHz) of compound **3** in $CDCl_3$. 13
Figure S22. NOESY spectrum (500 MHz) of compound **3** in $CDCl_3$. 13
Figure S23. IR spectrum of compound **3**. 14
Figure S24. HR-ESIMS spectrum of compound **3**. 14
Figure S25. ^{1}H NMR spectrum (500 MHz) of compound **4** in $CDCl_3$. 15
Figure S26. ^{13}C NMR spectrum (125 MHz) of compound **4** in $CDCl_3$. 15
Figure S27. HSQC spectrum (500 MHz) of compound **4** in $CDCl_3$. 16
Figure S28. HMBC spectrum (500 MHz) of compound **4** in $CDCl_3$. 16
Figure S29. COSY spectrum (500 MHz) of compound **4** in $CDCl_3$. 17
Figure S30. NOESY spectrum (500 MHz) of compound **4** in $CDCl_3$. 17
Figure S31. IR spectrum of compound **4**. 18
Figure S32. HR-ESIMS spectrum of compound **4**. 18
Figure S33. ^{1}H NMR spectrum (500 MHz) of compound **5** in $CDCl_3$. 19
Figure S34. ^{13}C NMR spectrum (125 MHz) of compound **5** in $CDCl_3$. 19
Figure S35. HSQC spectrum (500 MHz) of compound **5** in $CDCl_3$. 20
Figure S36. HMBC spectrum (500 MHz) of compound **5** in $CDCl_3$. 20
Figure S37. COSY spectrum (500 MHz) of compound **5** in $CDCl_3$. 21
Figure S38. NOESY spectrum (500 MHz) of compound **5** in $CDCl_3$. 21
Figure S39. IR spectrum of compound **5**. 22
Figure S40. HR-ESIMS spectrum of compound **5**. 22
Figure S41. ^{1}H NMR spectrum (500 MHz) of compound **6** in $CDCl_3$. 23

Figure S42. ^{13}C NMR spectrum (125 MHz) of compound **6** in $CDCl_3$. 23
Figure S43. HSQC spectrum (500 MHz) of compound **6** in $CDCl_3$. 24
Figure S44. HMBC spectrum (500 MHz) of compound **6** in $CDCl_3$. 24
Figure S45. COSY spectrum (500 MHz) of compound **6** in $CDCl_3$. 25
Figure S46. NOESY spectrum (500 MHz) of compound **6** in $CDCl_3$. 25
Figure S47. IR spectrum of compound **6**. 26
Figure S48. HR-ESIMS spectrum of compound **6**. 26

Figure S1. ^{1}H NMR spectrum (500 MHz) of compound **1** in $CDCl_3$.

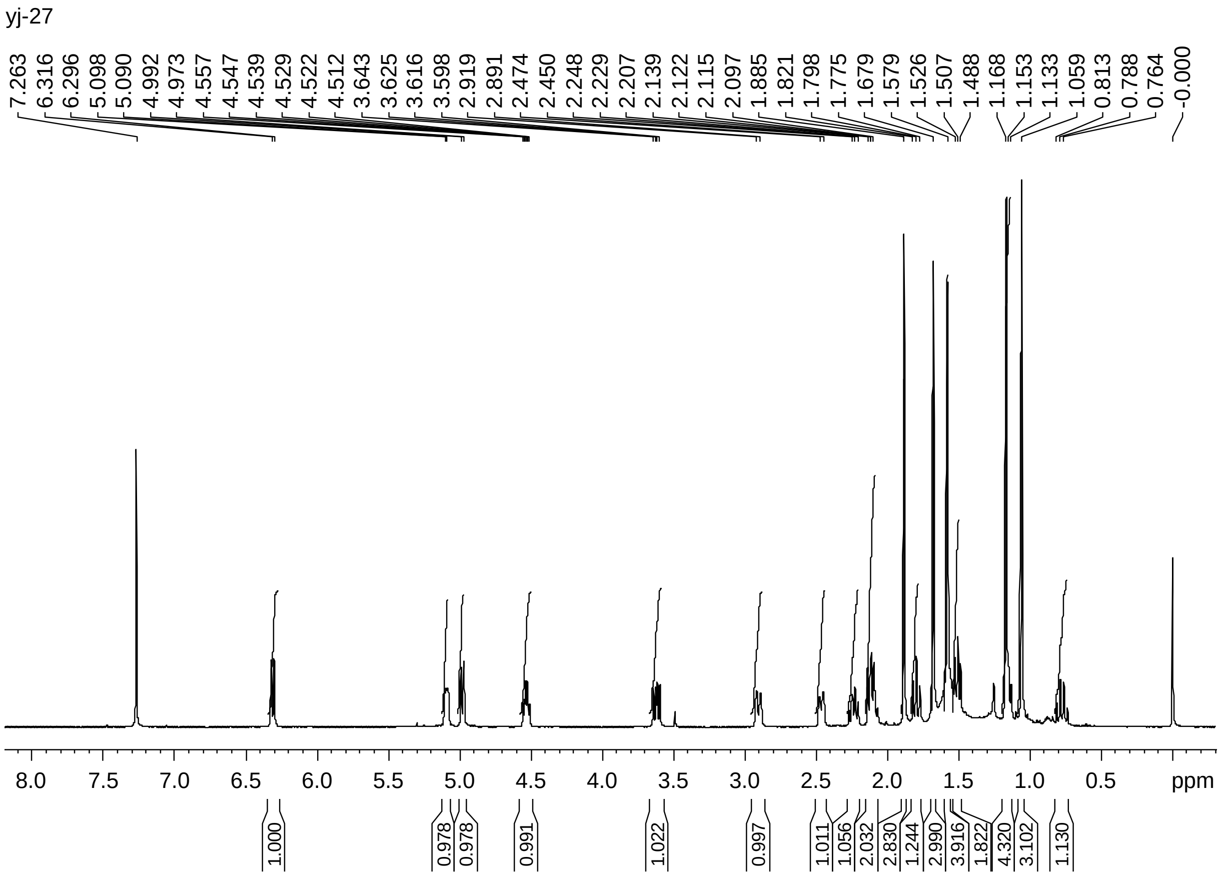

Figure S2. ^{13}C NMR spectrum (125 MHz) of compound **1** in $CDCl_3$.

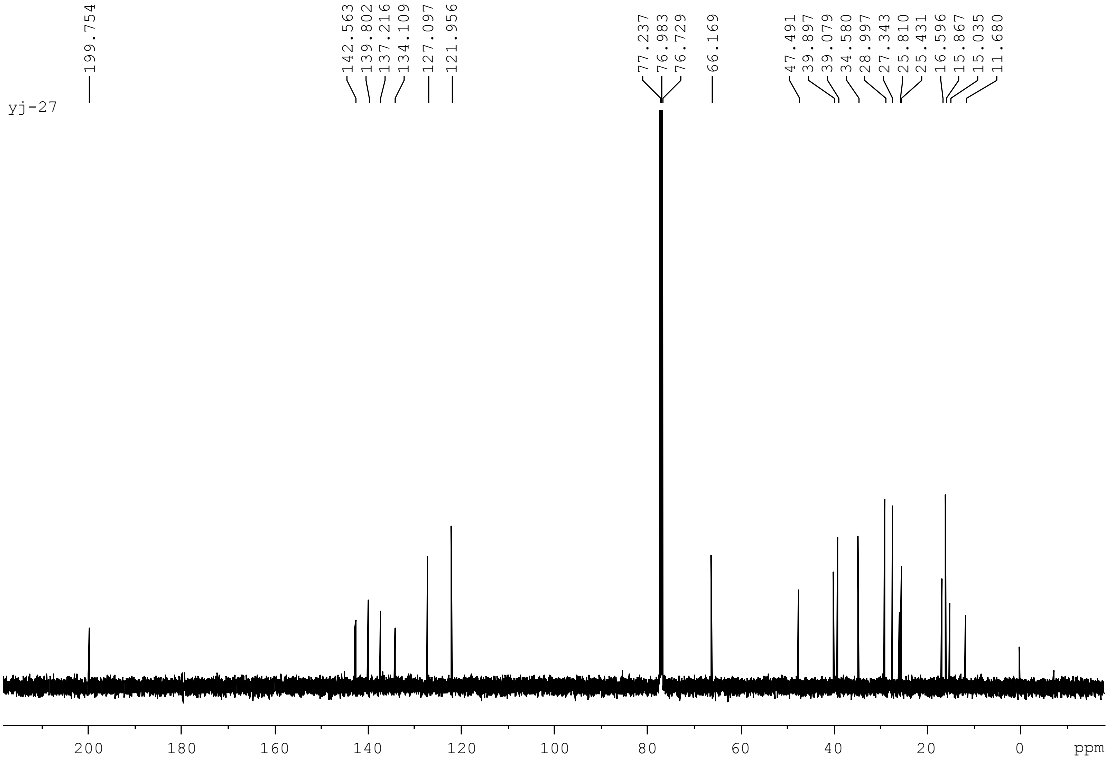

Figure S3. HSQC spectrum (500 MHz) of compound **1** in $CDCl_3$.

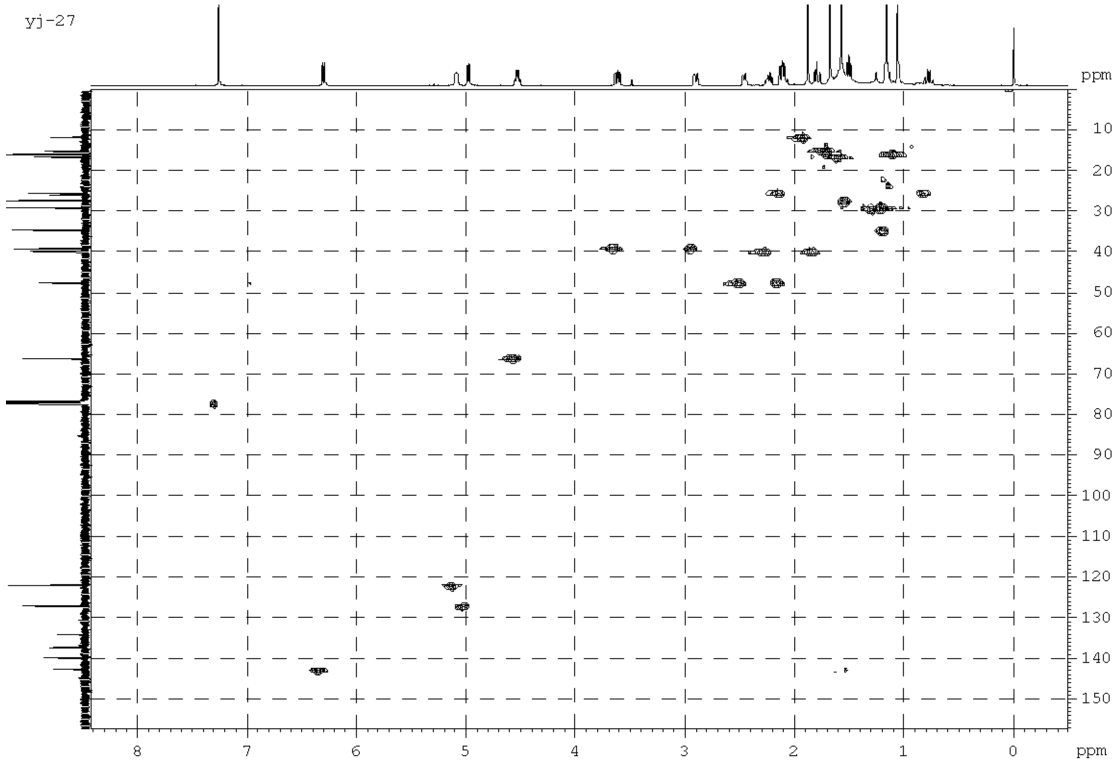

Figure S4. HMBC spectrum (500 MHz) of compound **1** in $CDCl_3$.

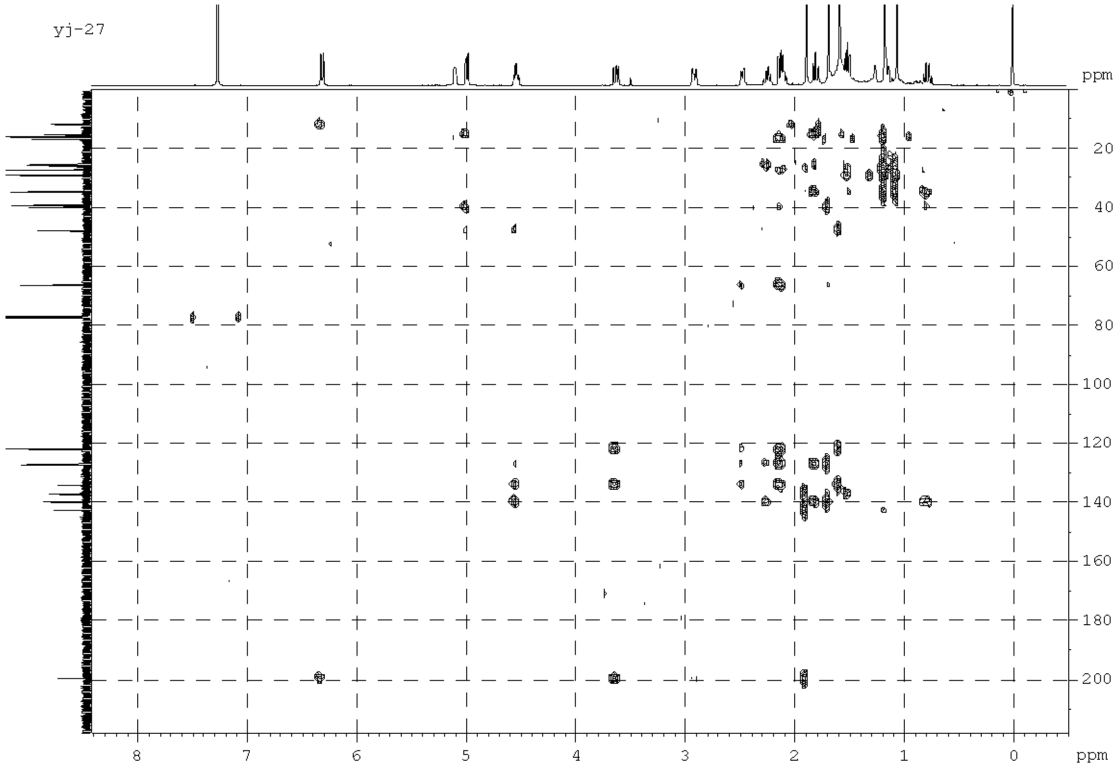

Figure S5. COSY spectrum (500 MHz) of compound **1** in $CDCl_3$.

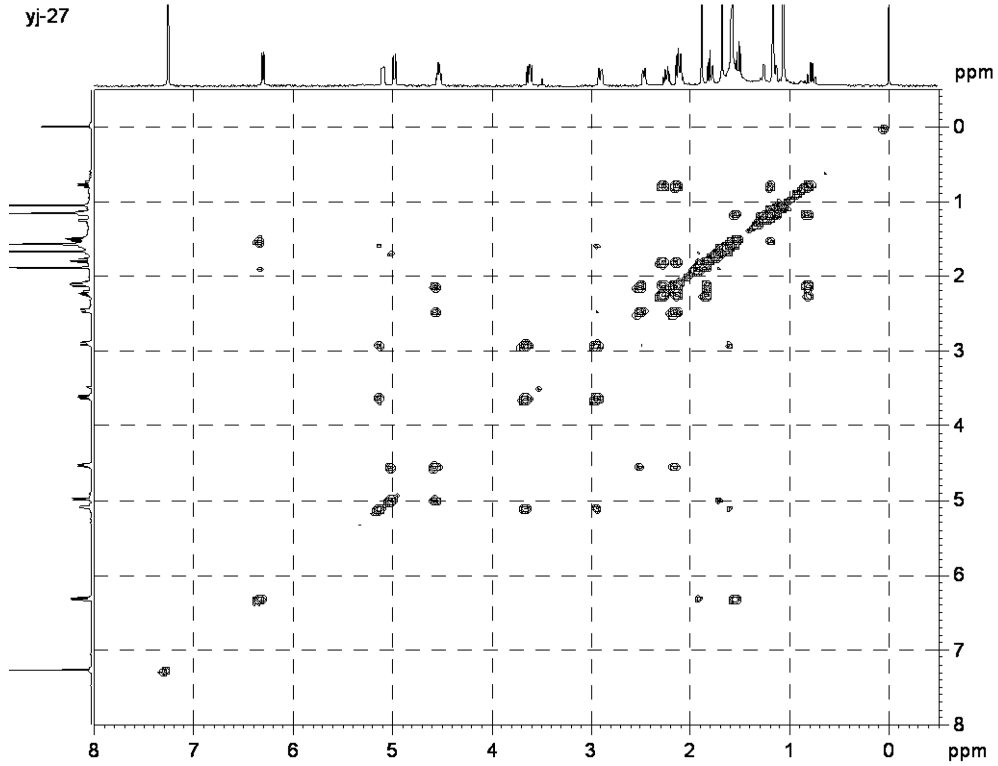

Figure S6. NOESY spectrum (500 MHz) of compound **1** in $CDCl_3$.

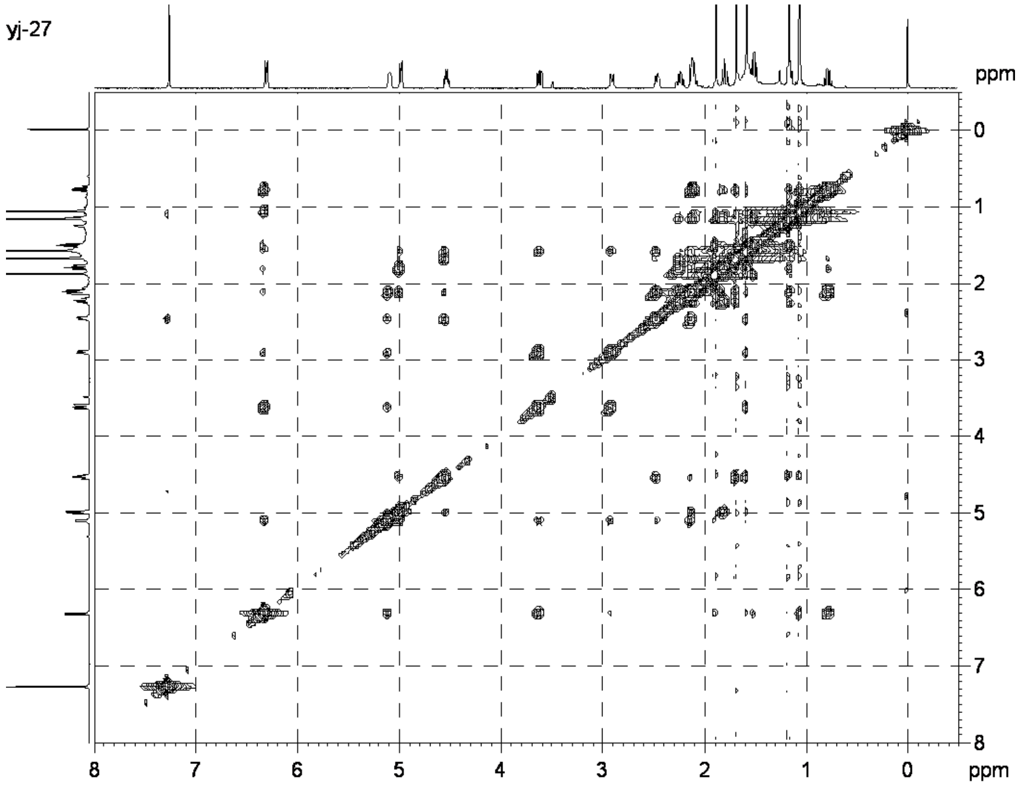

Figure S7. IR spectrum of compound **1**.

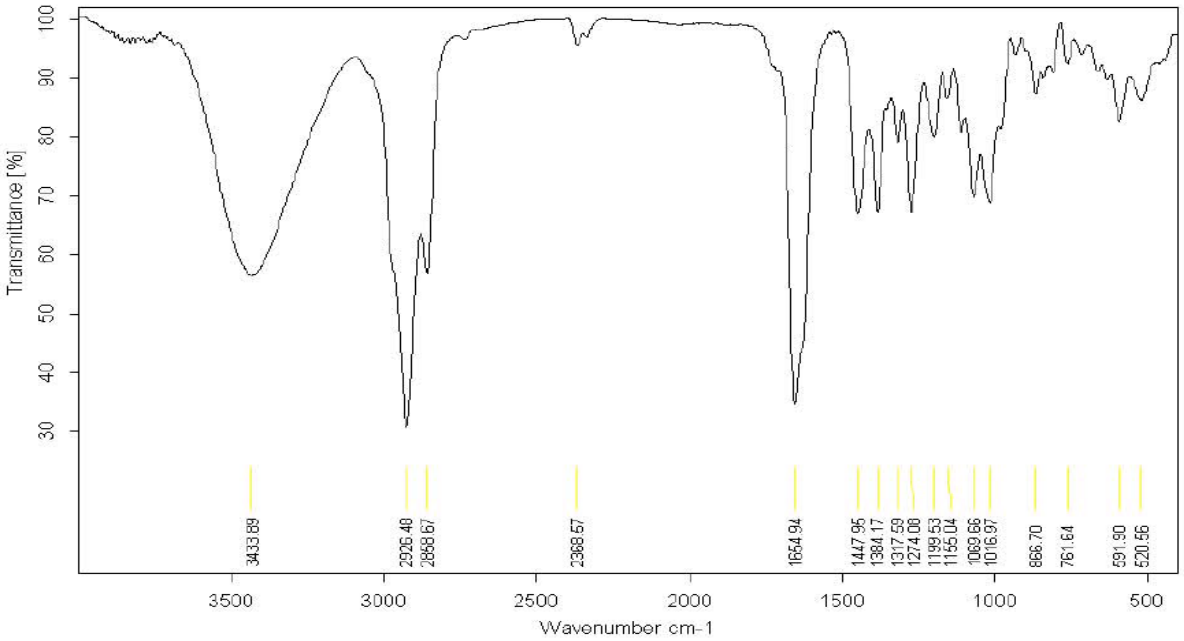

Figure S8. HR-ESIMS spectrum of compound **1**.

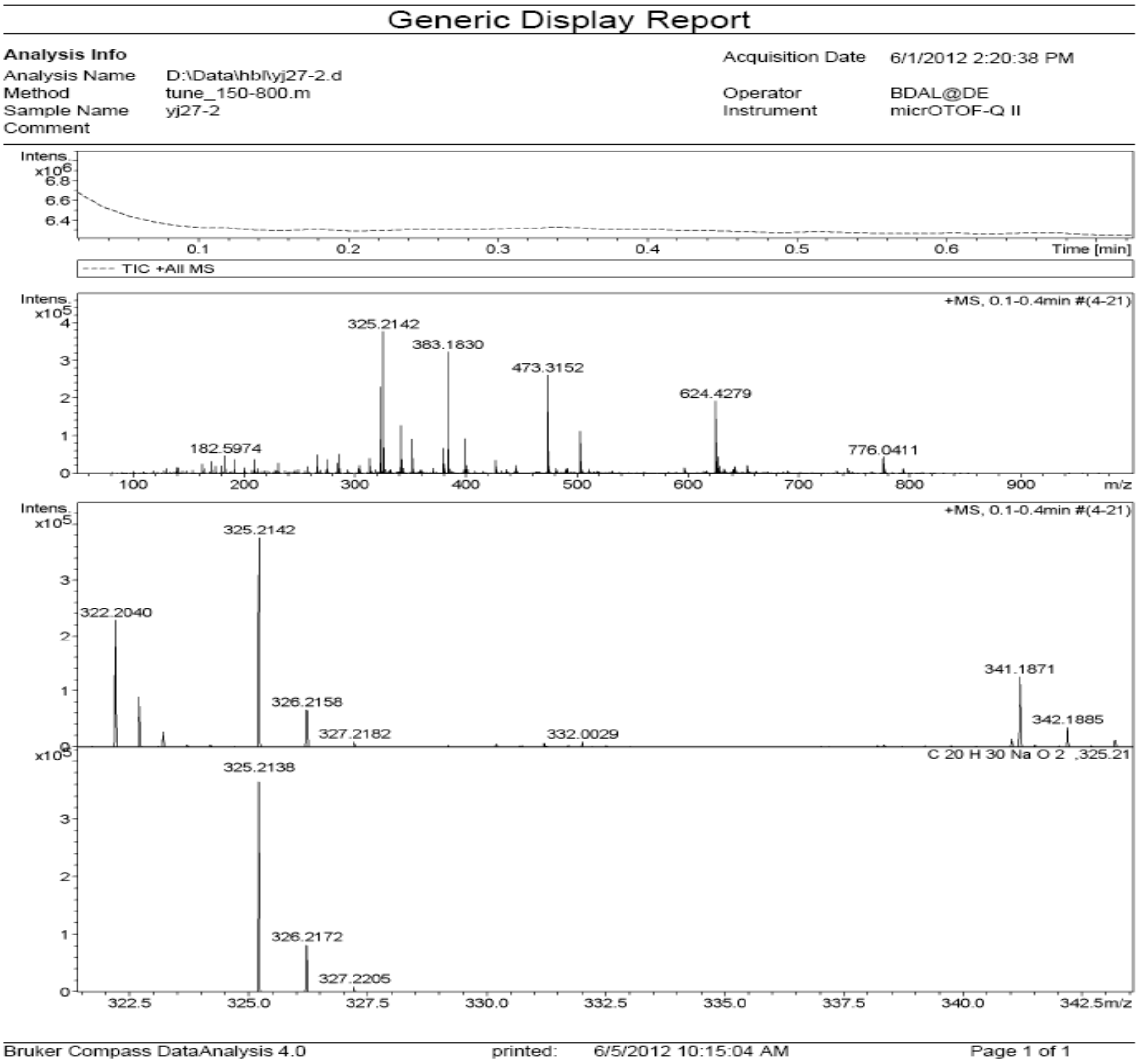

Figure S9. ^{1}H NMR spectrum (500 MHz) of compound **2** in $CDCl_3$.

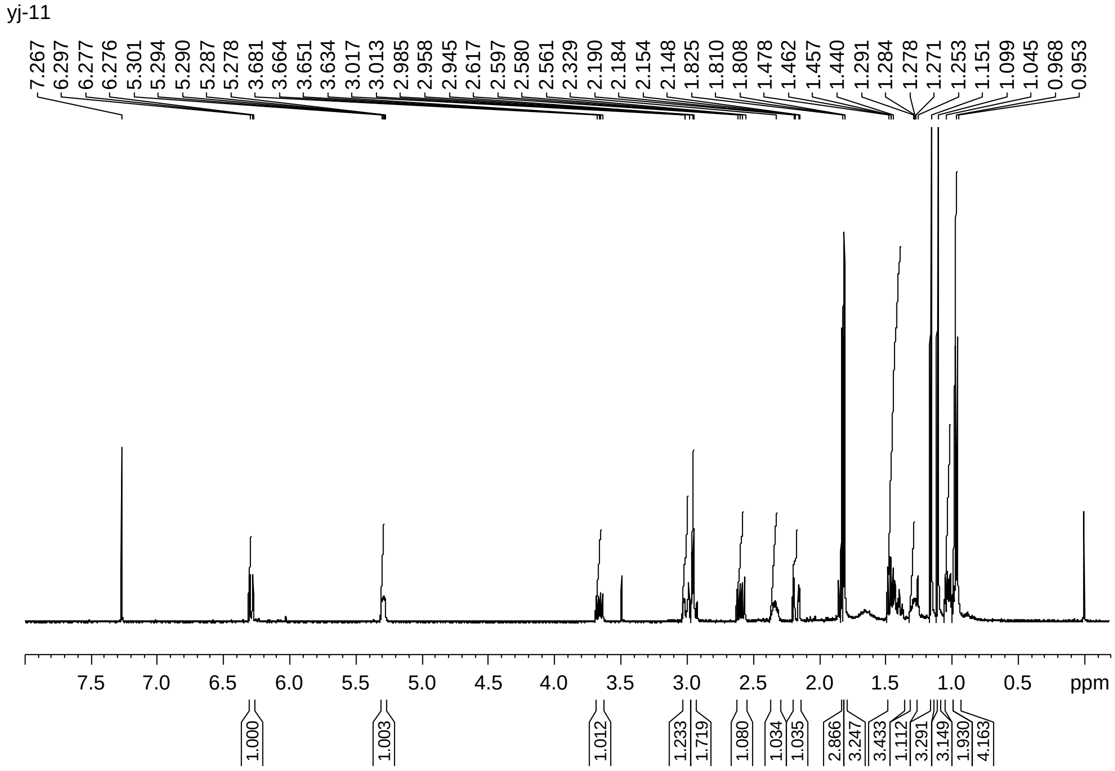

Figure S10. ^{13}C NMR spectrum (125 MHz) of compound **2** in $CDCl_3$.

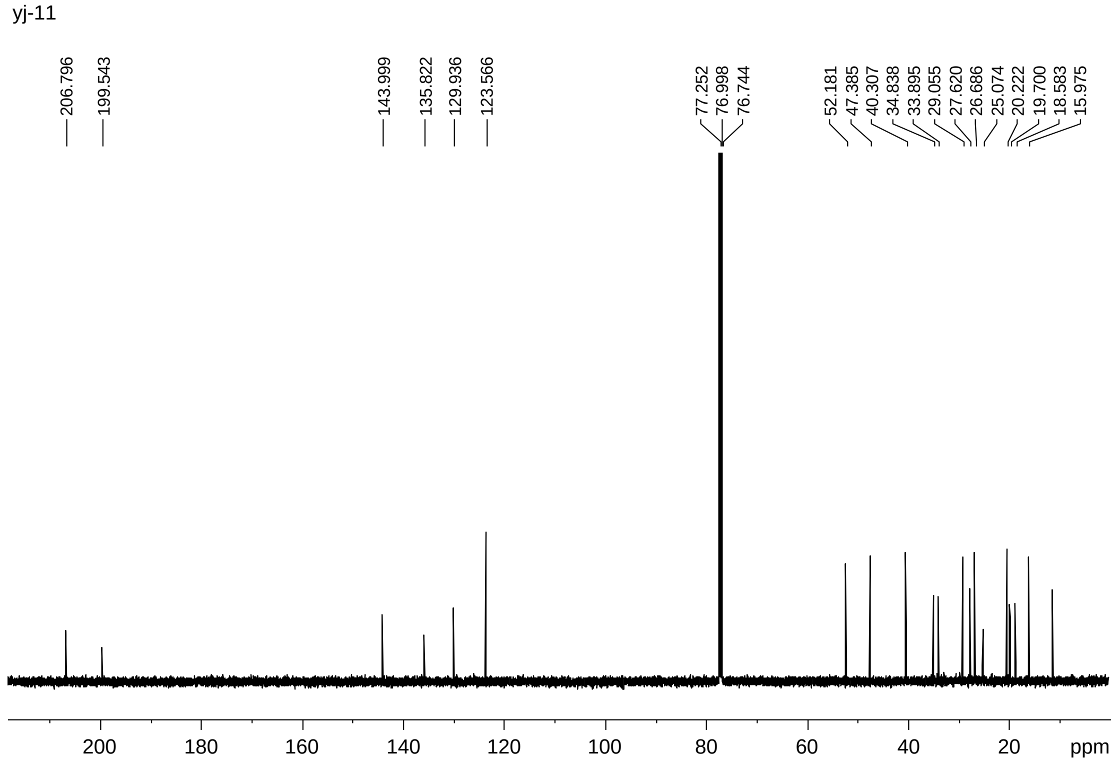

Figure S11. HSQC spectrum (500 MHz) of compound **2** in $CDCl_3$.

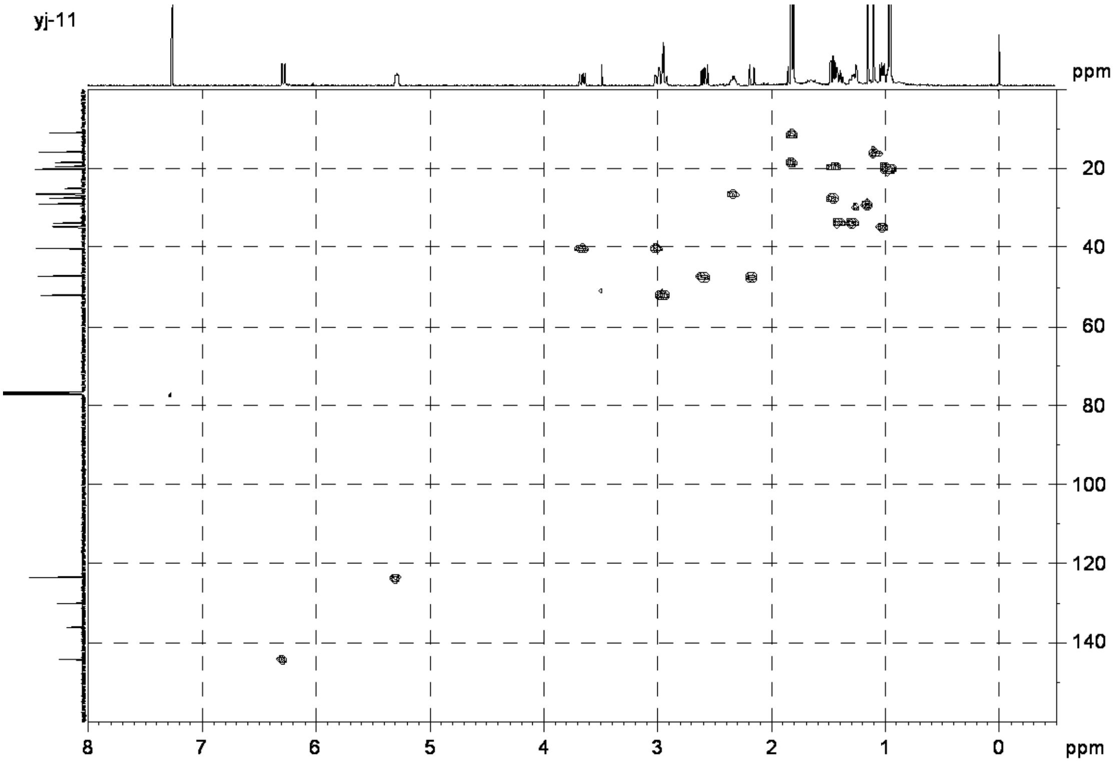

Figure S12. HMBC spectrum (500 MHz) of compound **2** in $CDCl_3$.

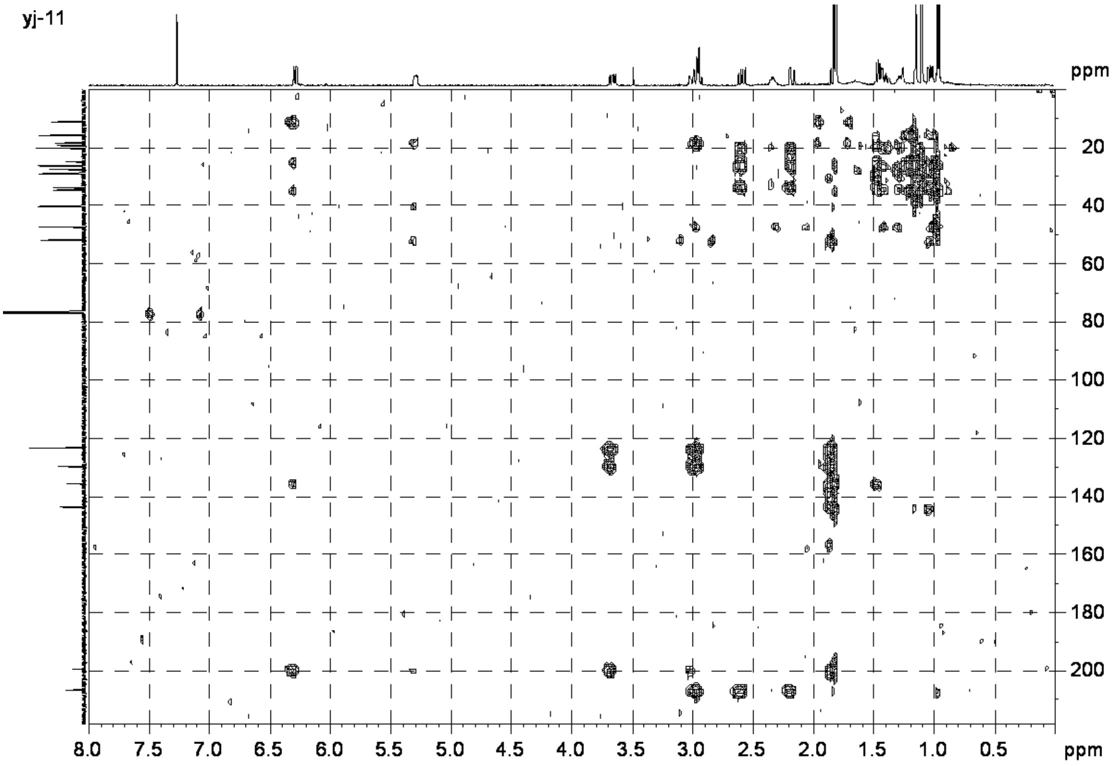

Figure S13. COSY spectrum (500 MHz) of compound **2** in $CDCl_3$.

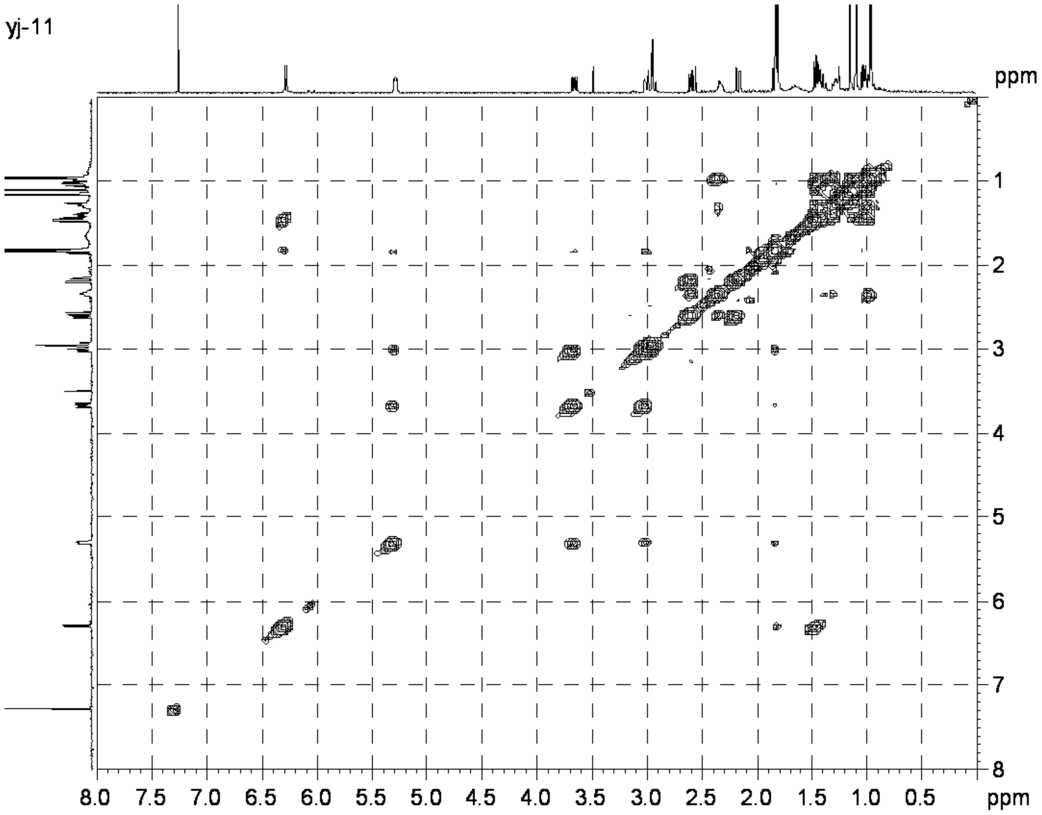

Figure S14. NOESY spectrum (500 MHz) of compound **2** in $CDCl_3$.

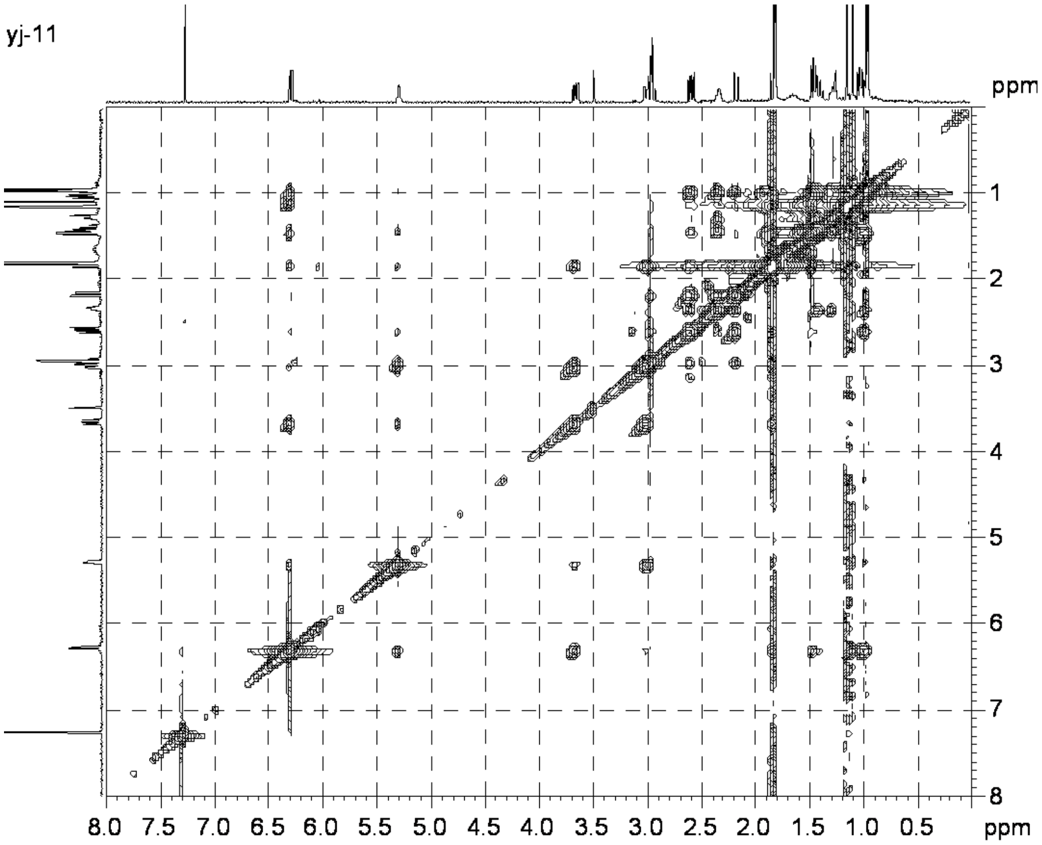

Figure S15. IR spectrum of compound **2**.

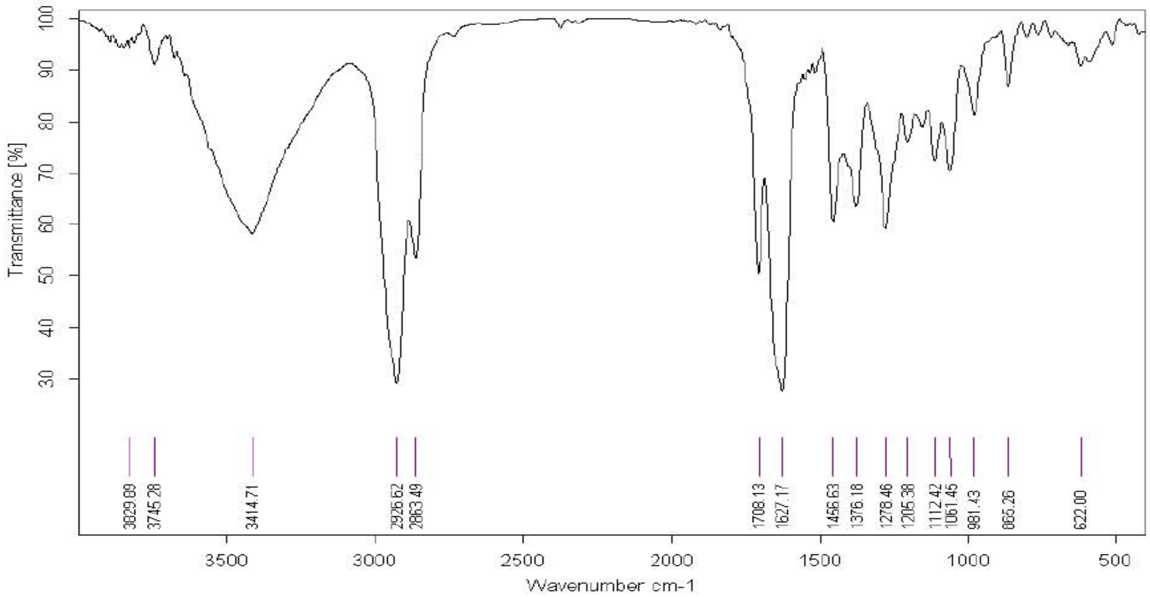

Figure S16. HR-ESIMS spectrum of compound **2**.

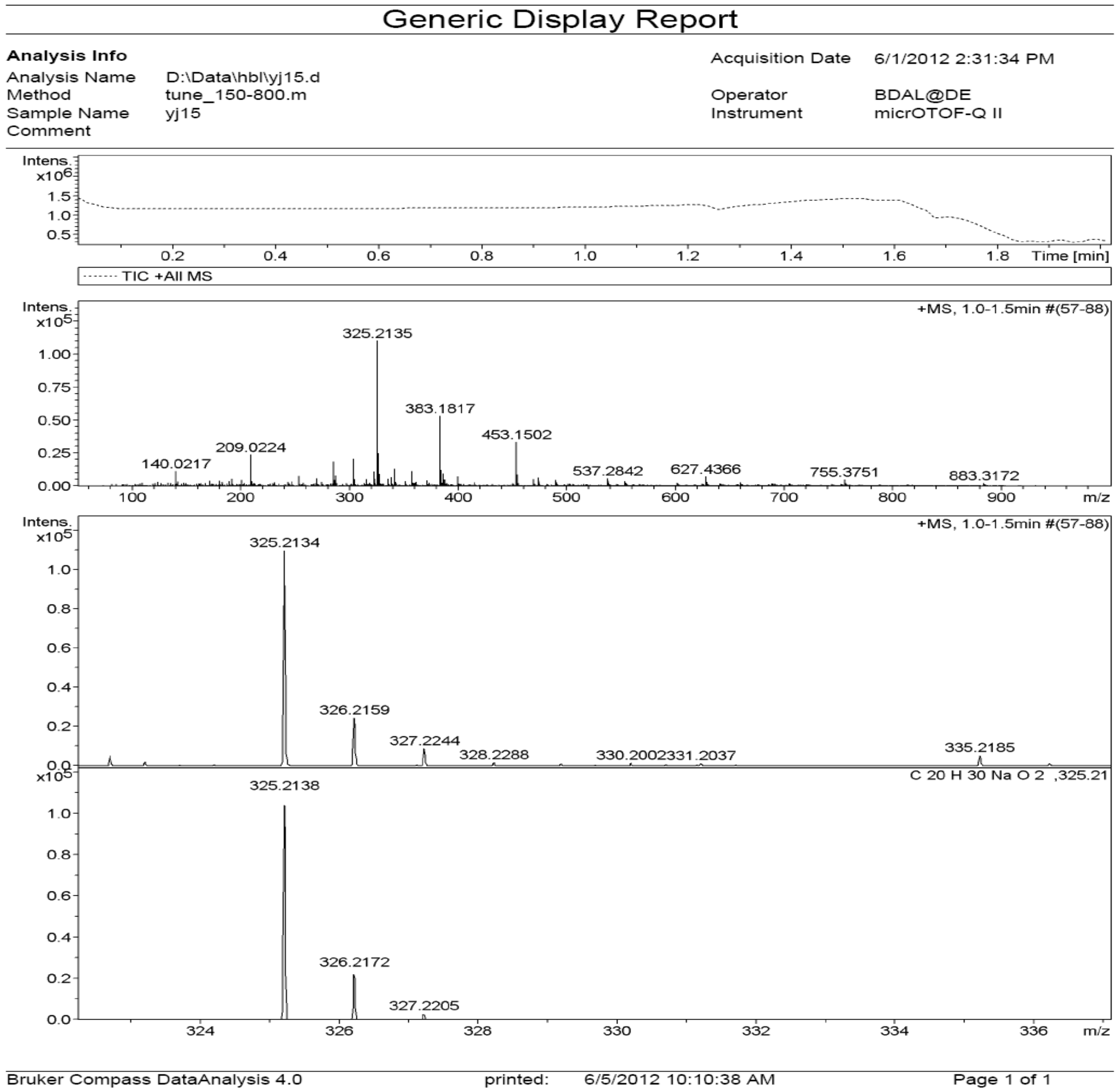

Figure S17. ^{1}H NMR spectrum (500 MHz) of compound **3** in $CDCl_3$.

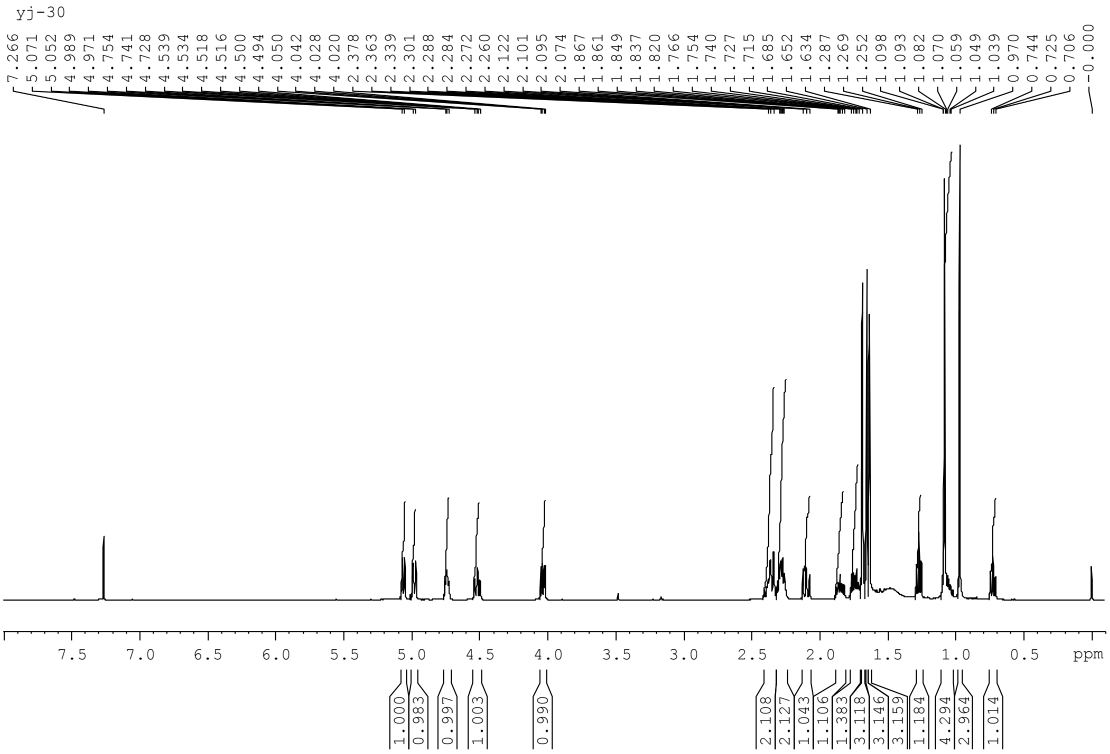

Figure S18. ^{13}C NMR spectrum (125 MHz) of compound **3** in $CDCl_3$.

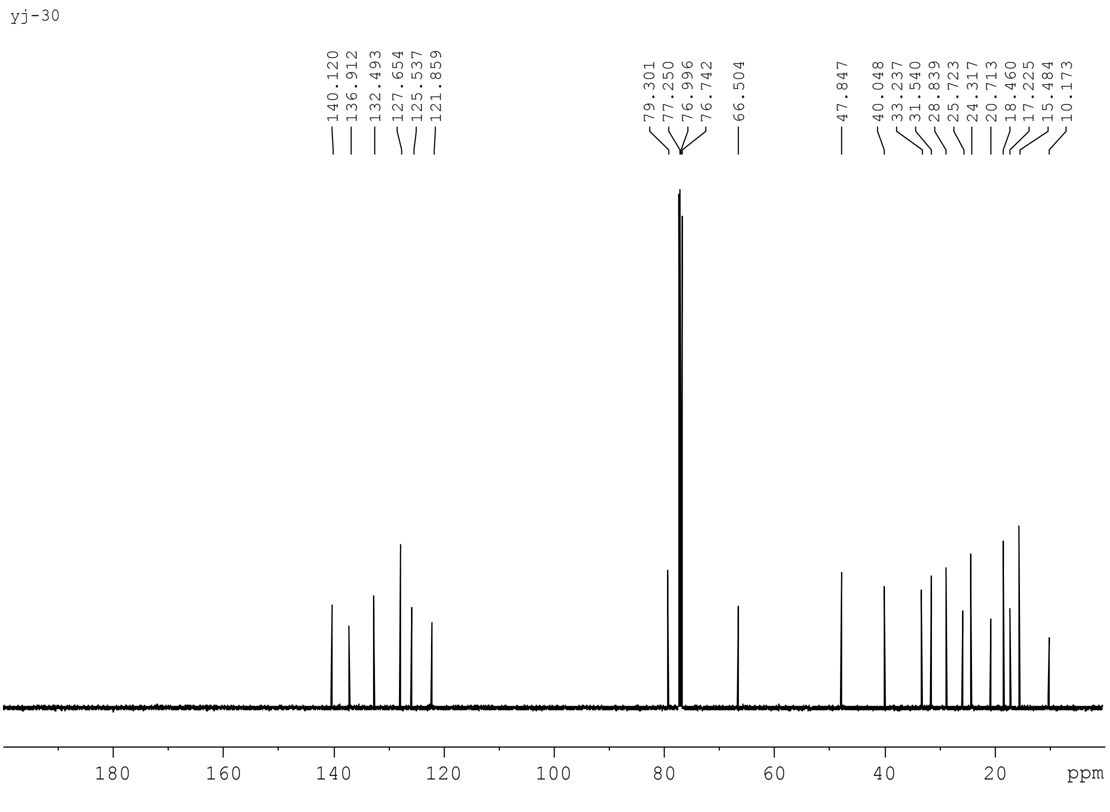

Figure S19. HSQC spectrum (500 MHz) of compound **3** in $CDCl_3$.

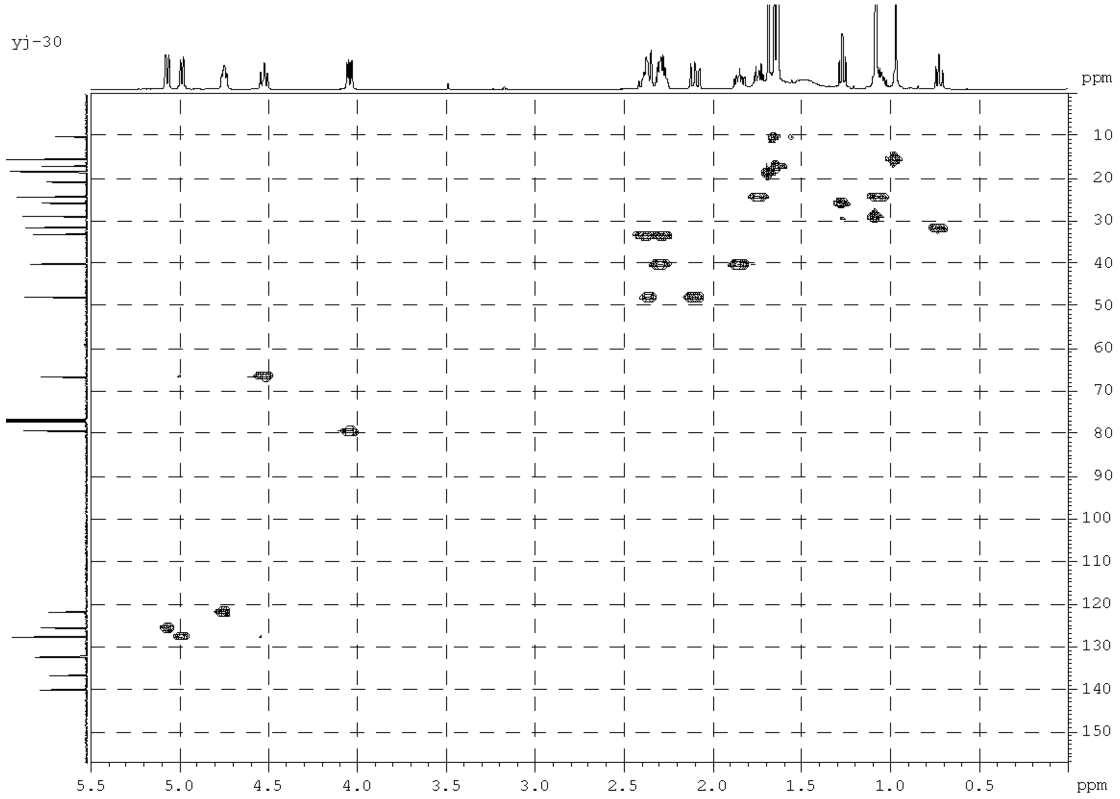

Figure S20. HMBC spectrum (500 MHz) of compound **3** in $CDCl_3$.

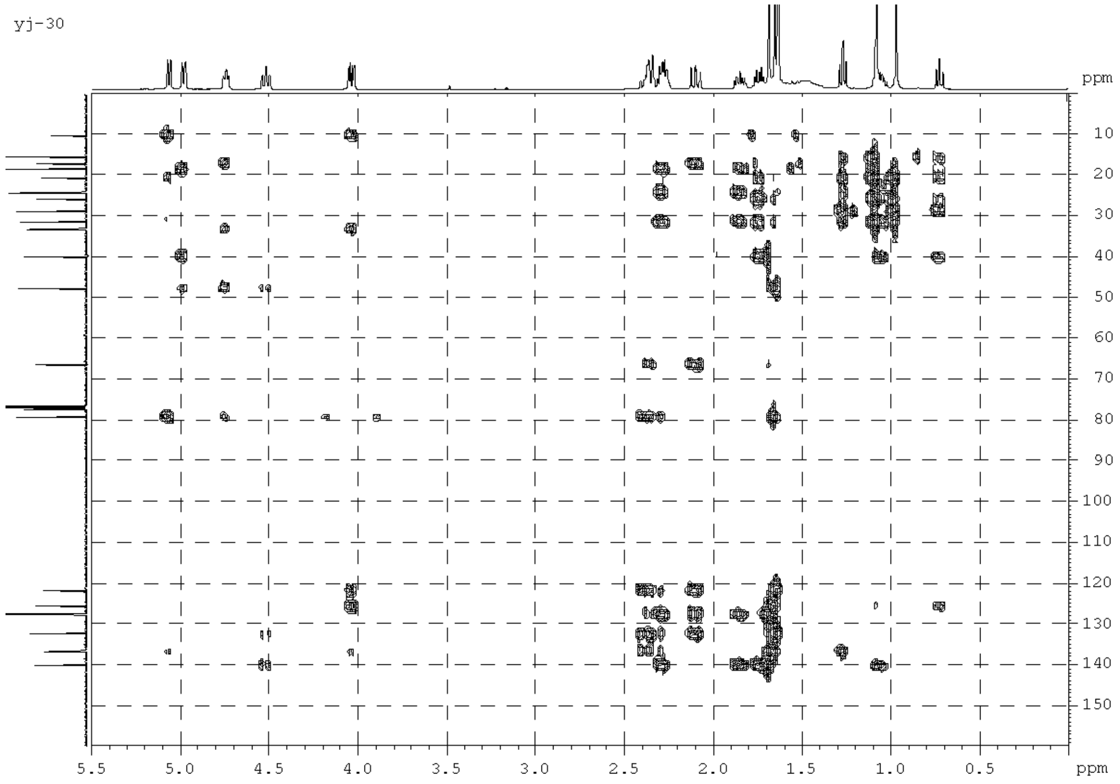

Figure S21. COSY spectrum (500 MHz) of compound **3** in $CDCl_3$.

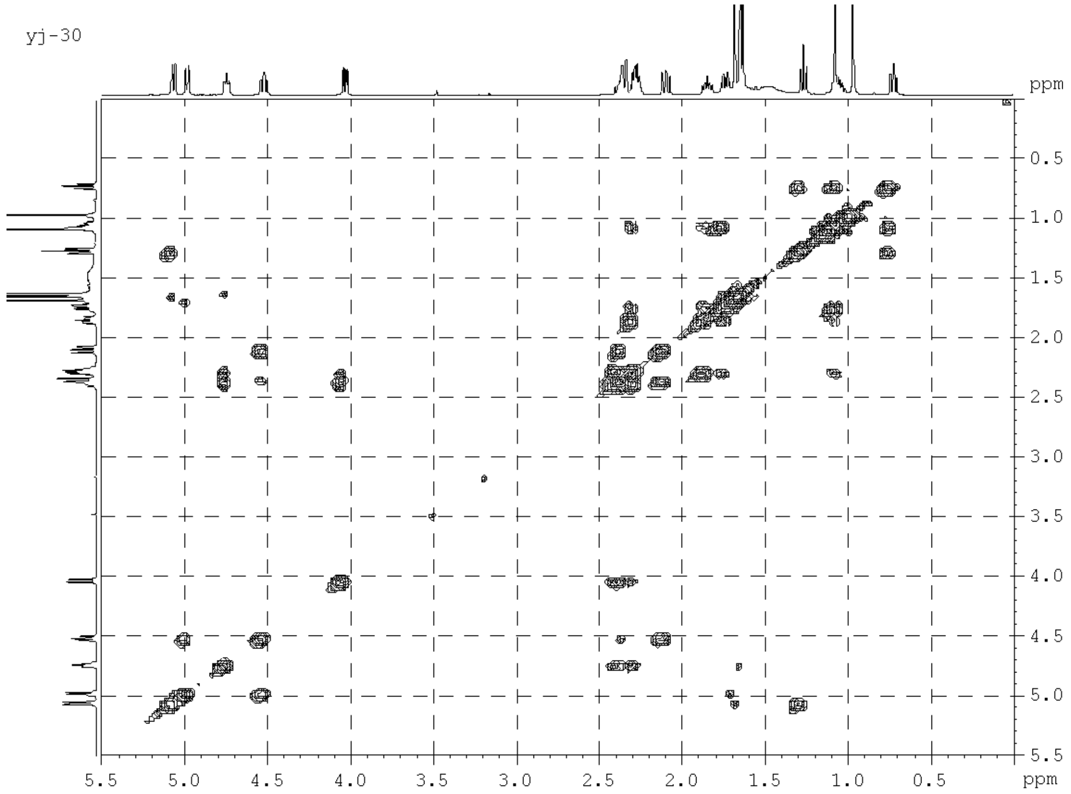

Figure S22. NOESY spectrum (500 MHz) of compound **3** in $CDCl_3$.

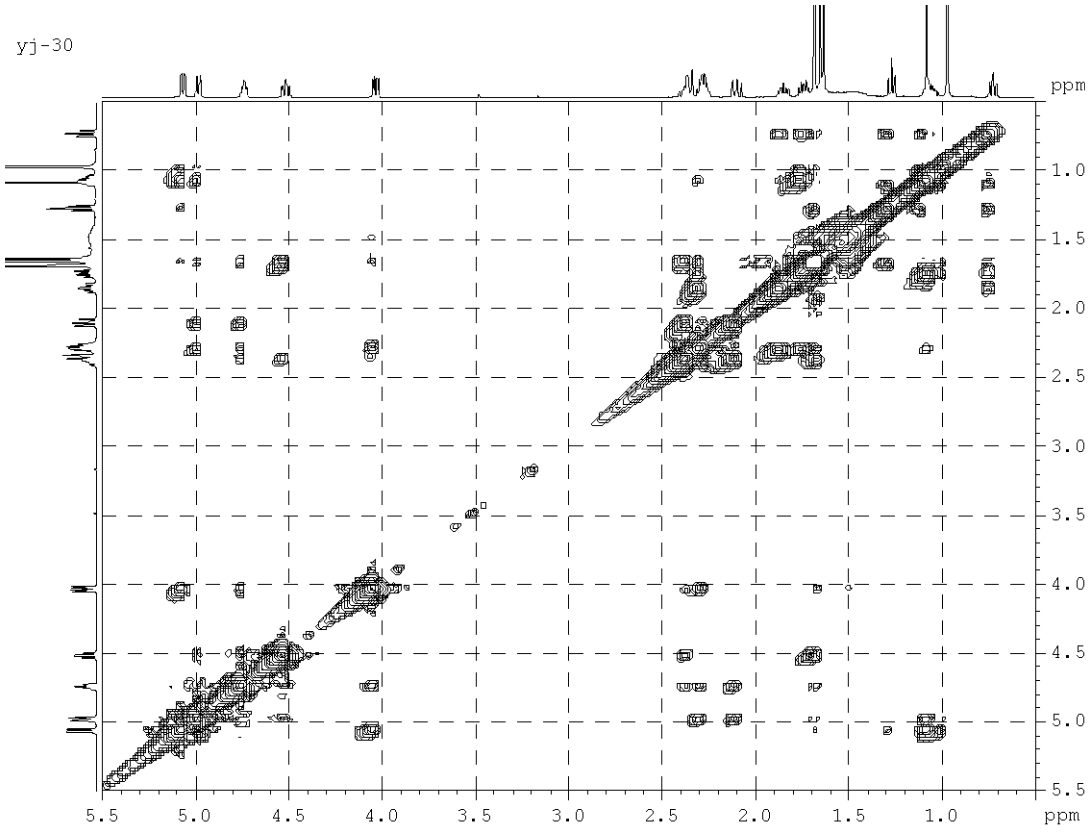

Figure S23. IR spectrum of compound **3**.

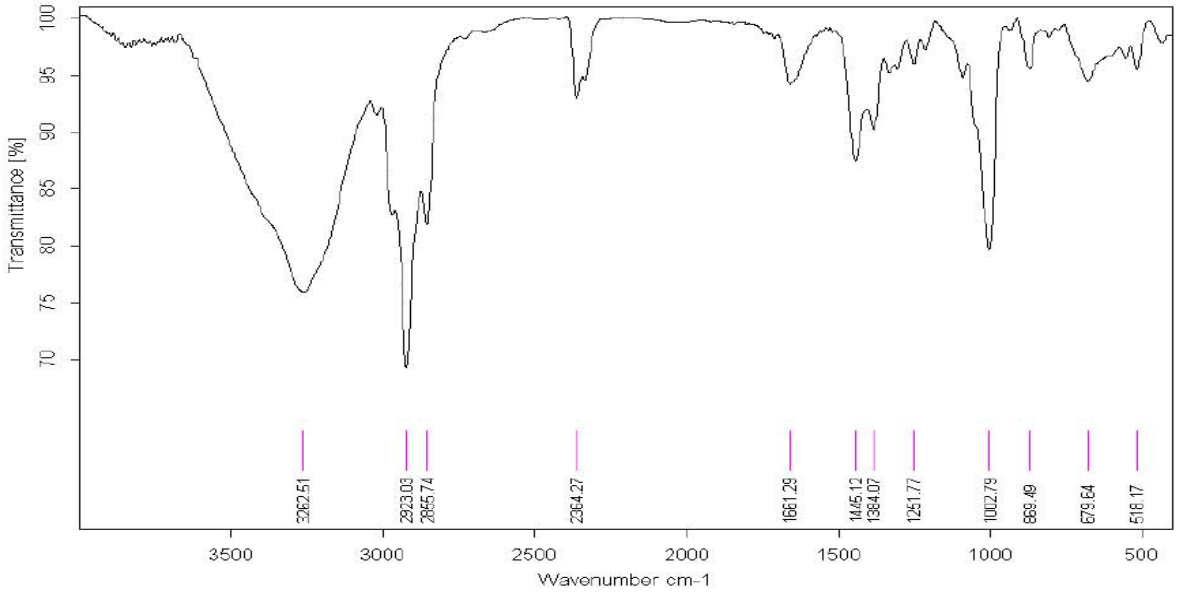

Figure S24. HR-ESIMS spectrum of compound **3**.

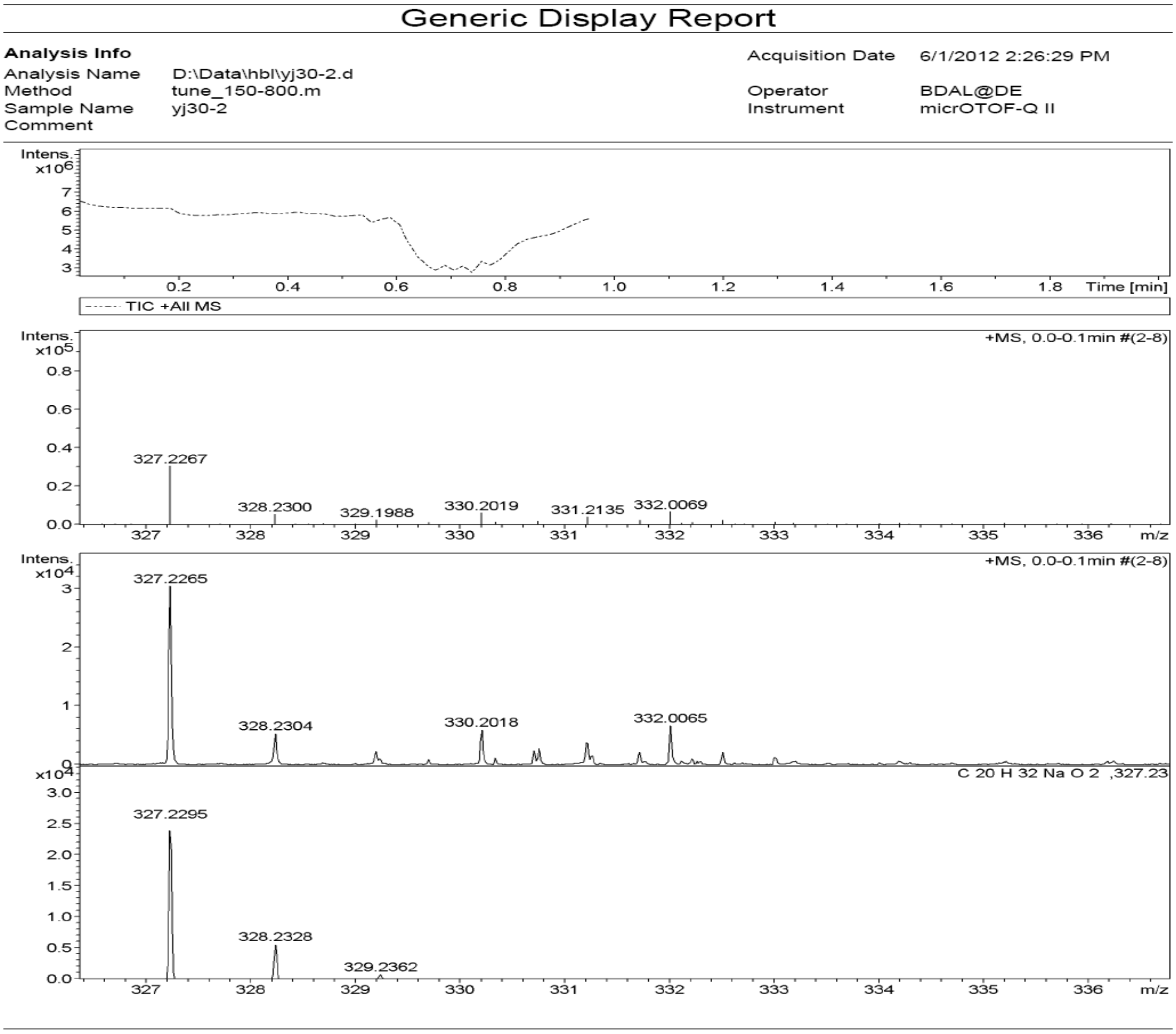

Figure S25. ^{1}H NMR spectrum (500 MHz) of compound **4** in $CDCl_3$.

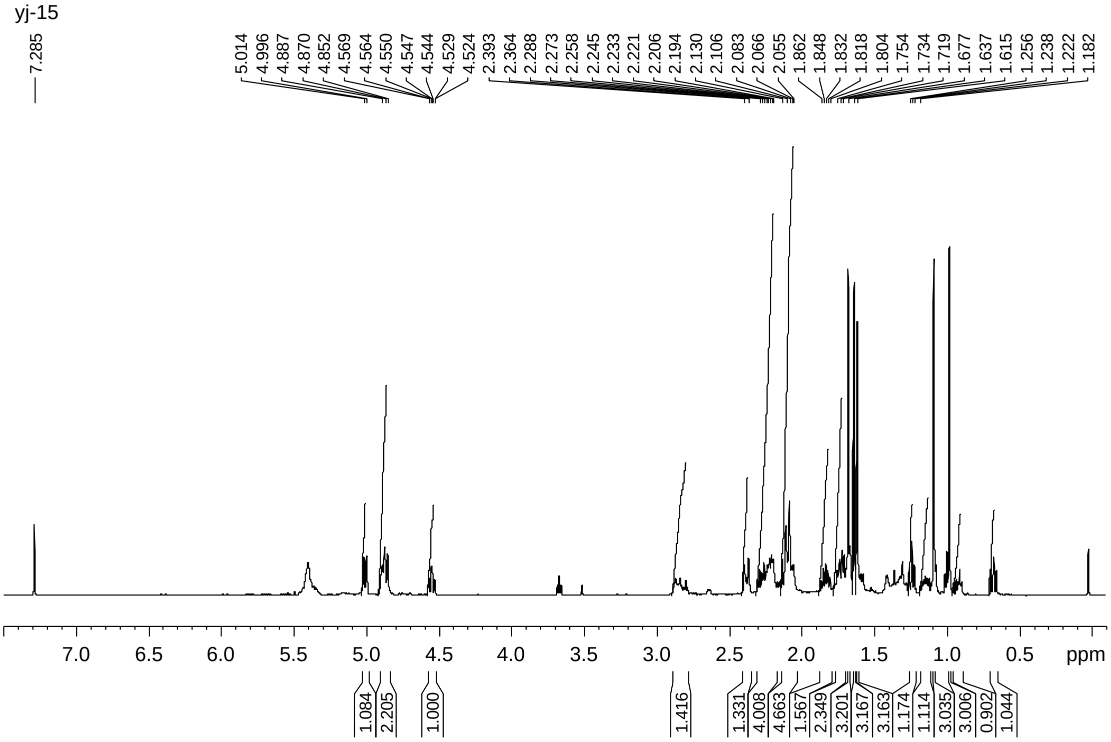

Figure S26. ^{13}C NMR spectrum (125 MHz) of compound **4** in $CDCl_3$.

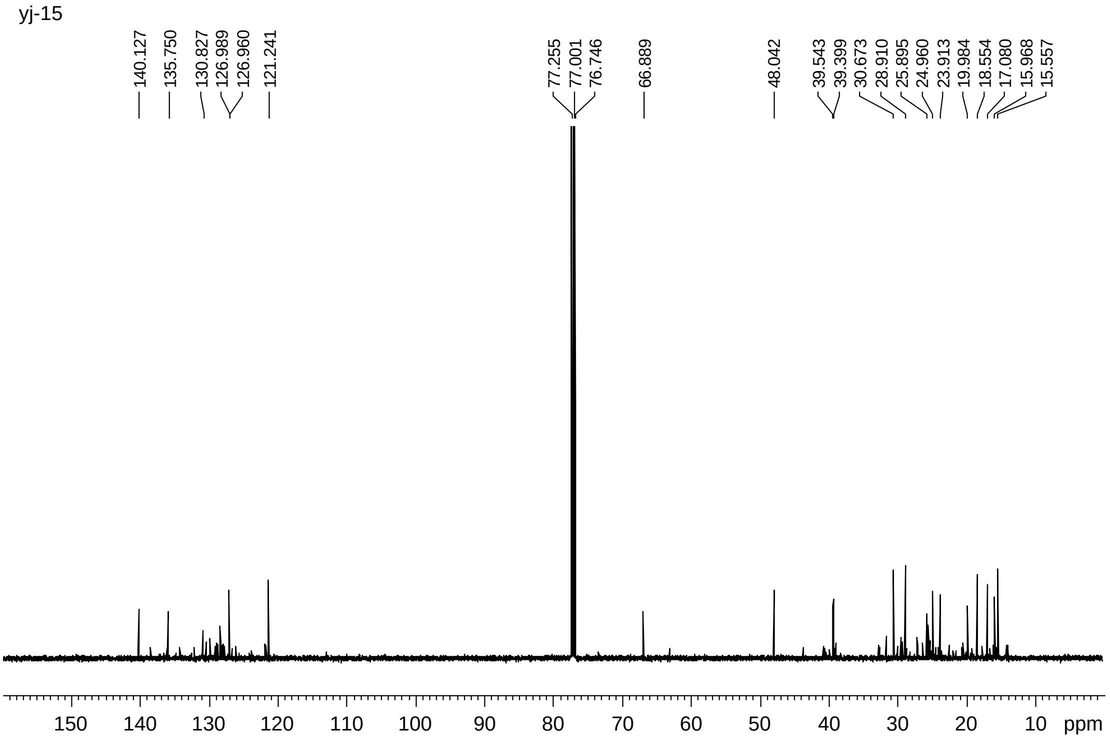

Figure S27. HSQC spectrum (500 MHz) of compound **4** in $CDCl_3$.

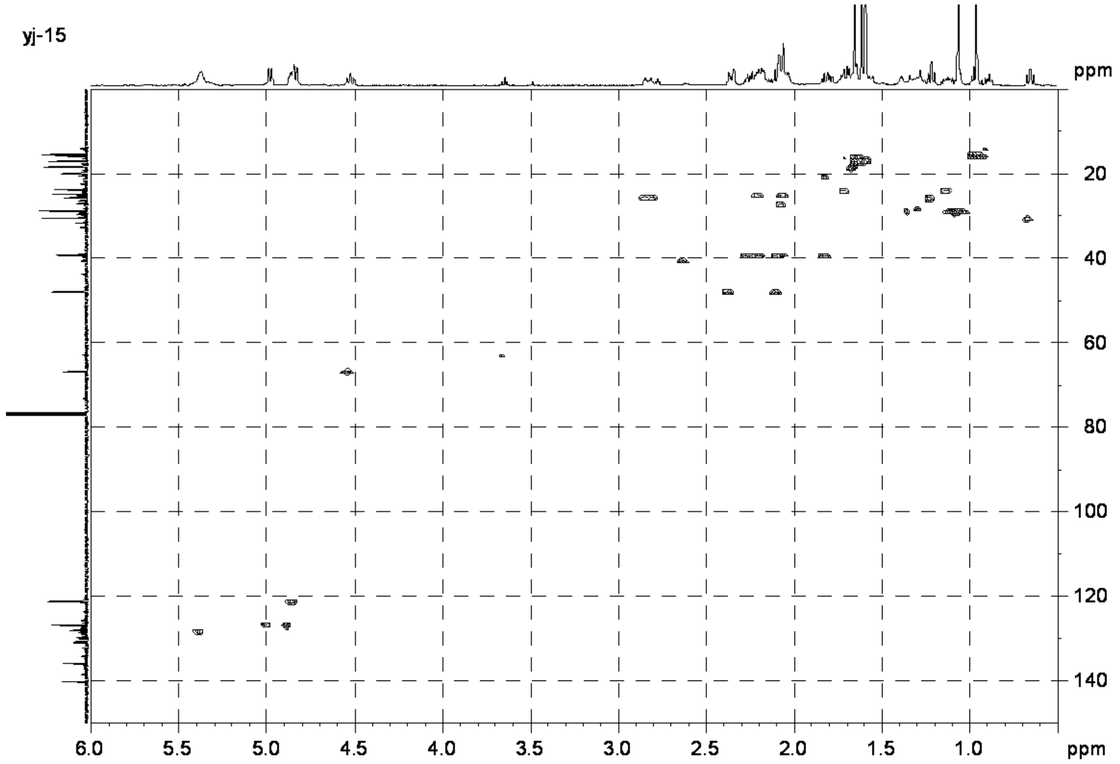

Figure S28. HMBC spectrum (500 MHz) of compound **4** in $CDCl_3$.

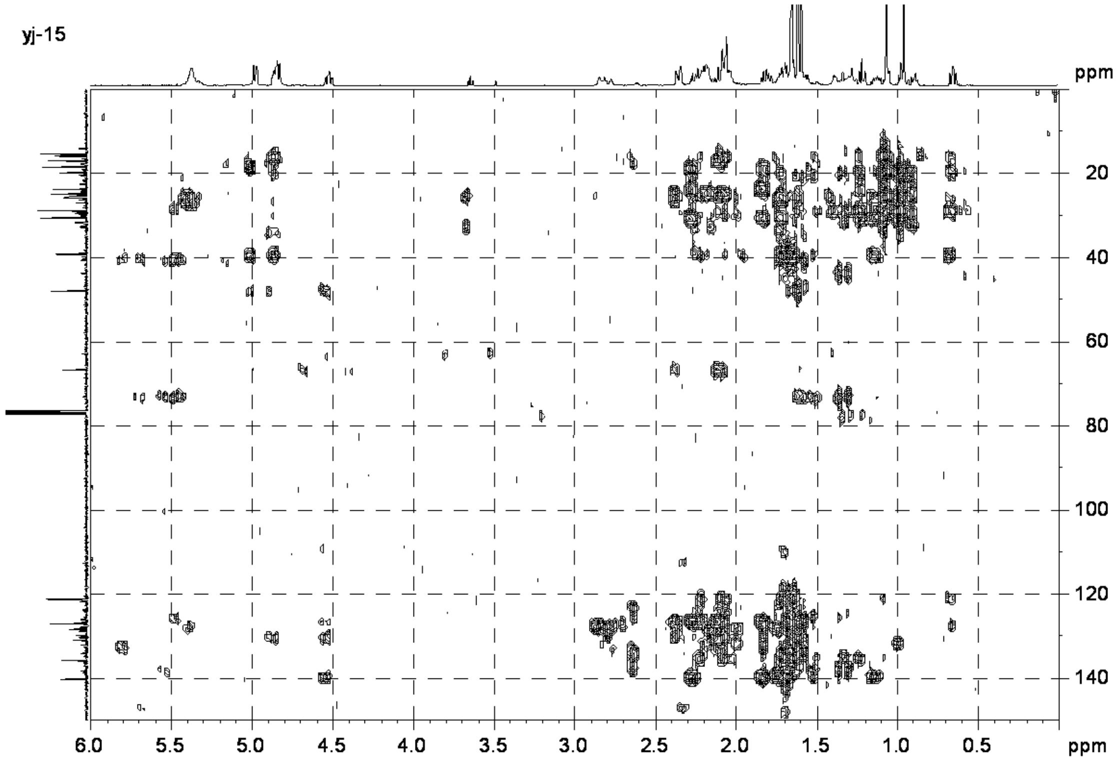

Figure S29. COSY spectrum (500 MHz) of compound **4** in $CDCl_3$.

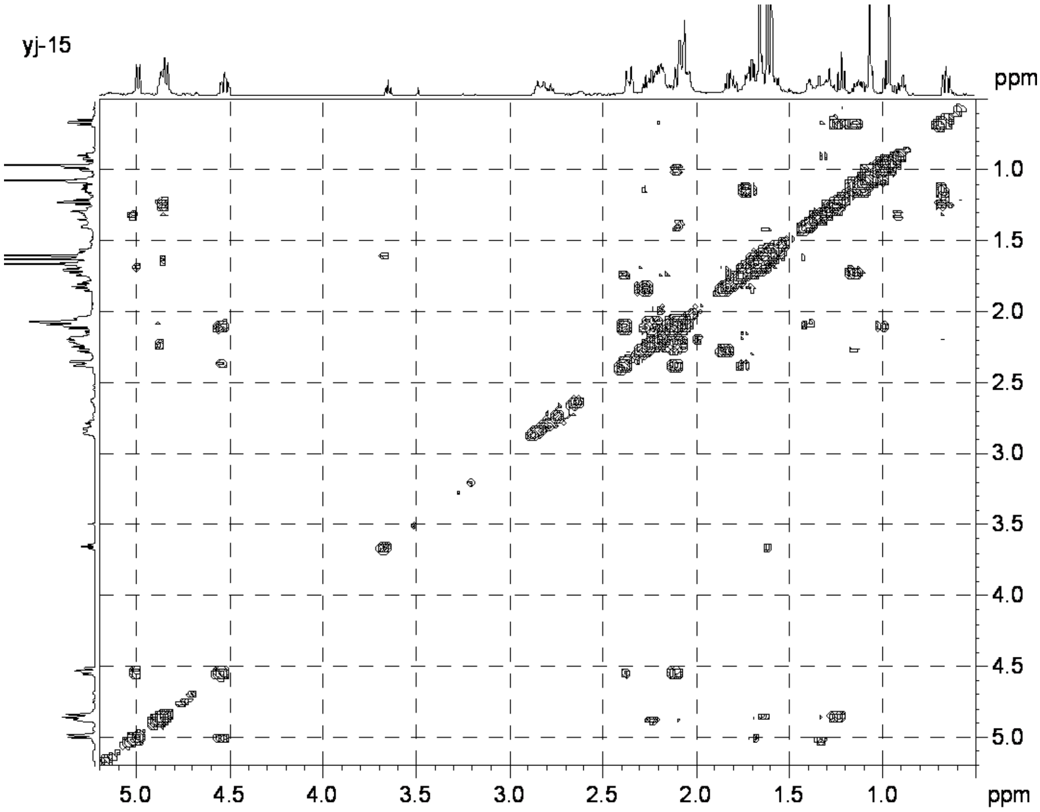

Figure S30. NOESY spectrum (500 MHz) of compound **4** in $CDCl_3$.

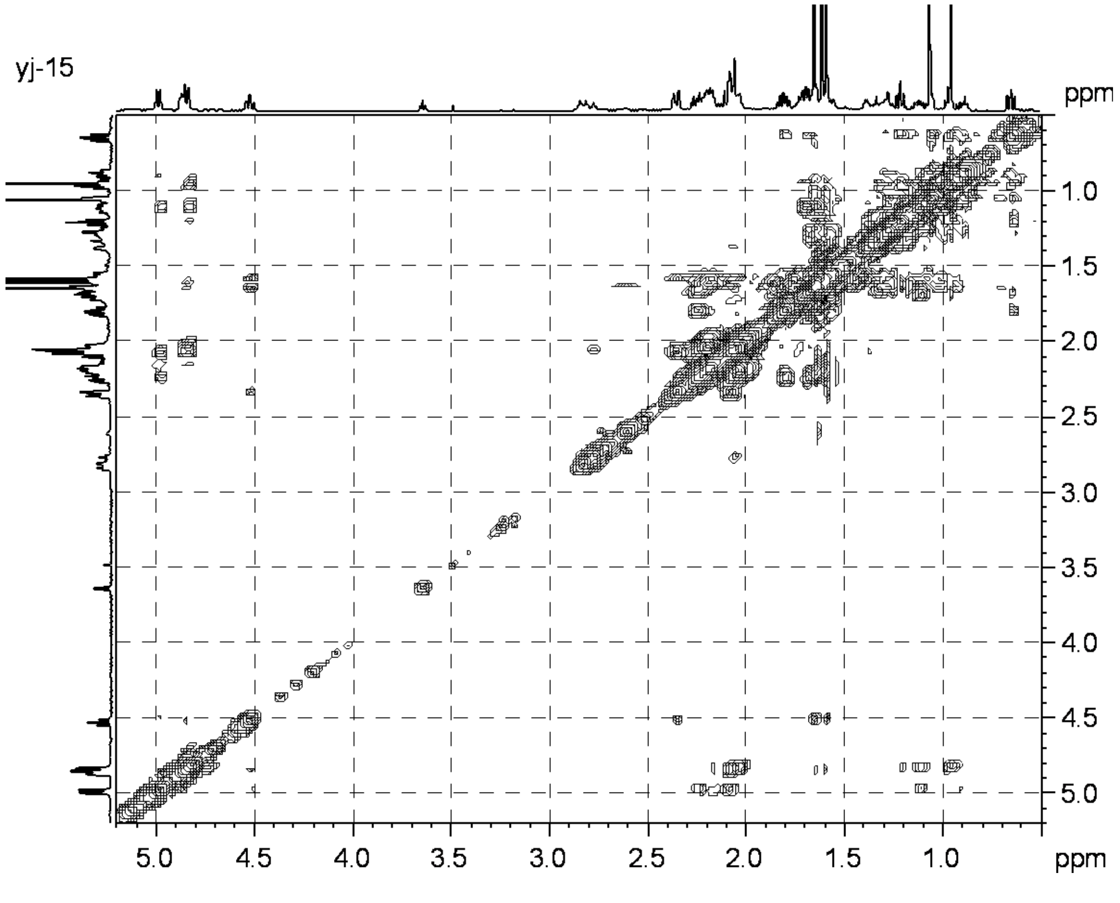

Figure S31. IR spectrum of compound **4**.

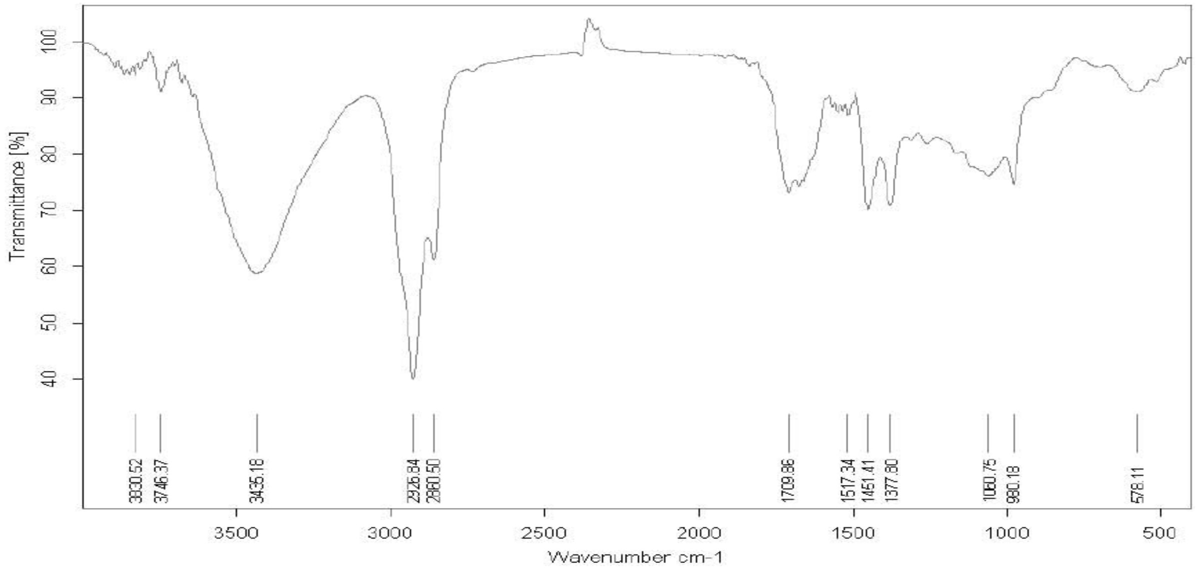

Figure S32. HR-ESIMS spectrum of compound **4**.

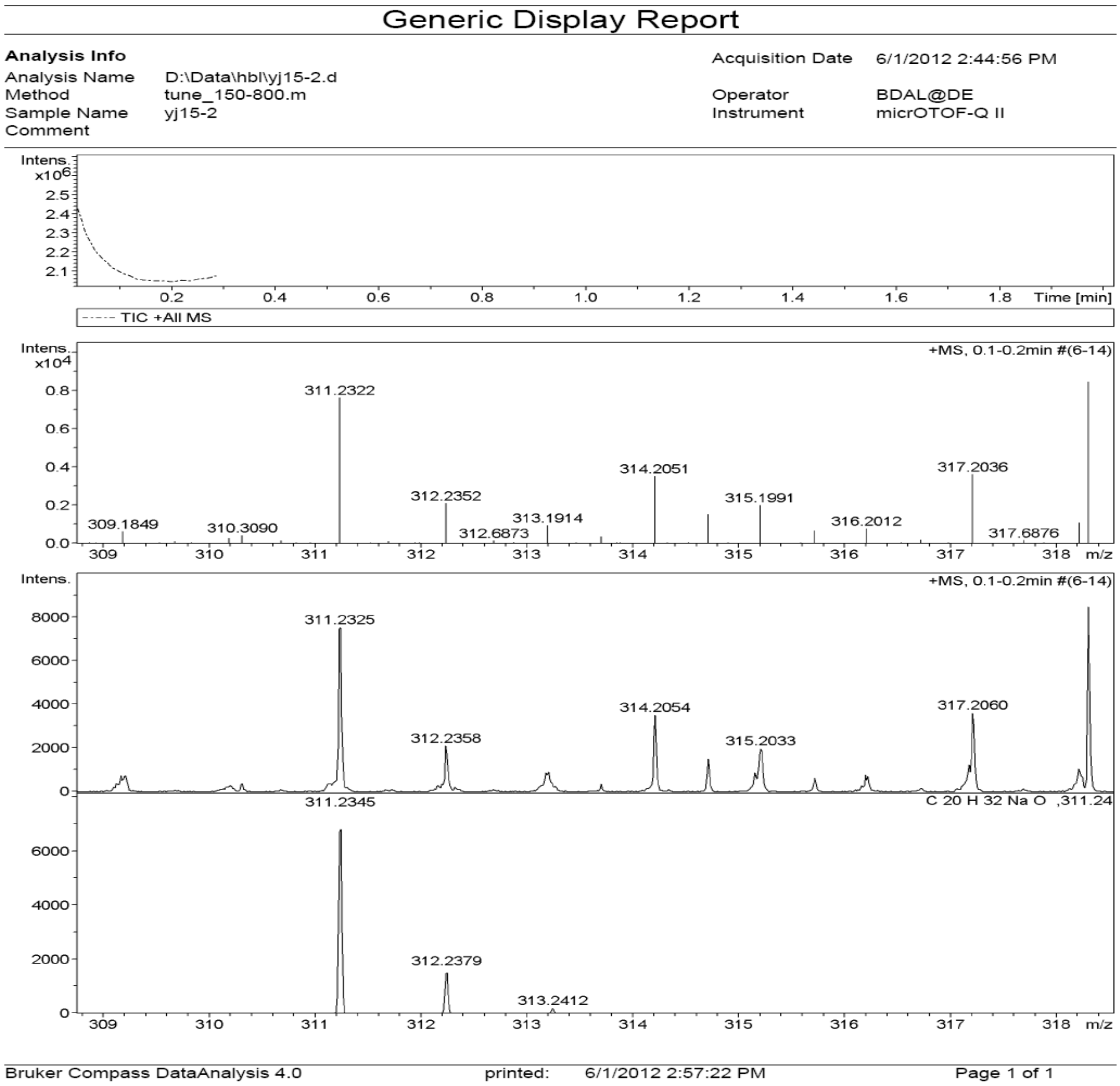

Figure S33. ^{1}H NMR spectrum (500 MHz) of compound **5** in $CDCl_3$.

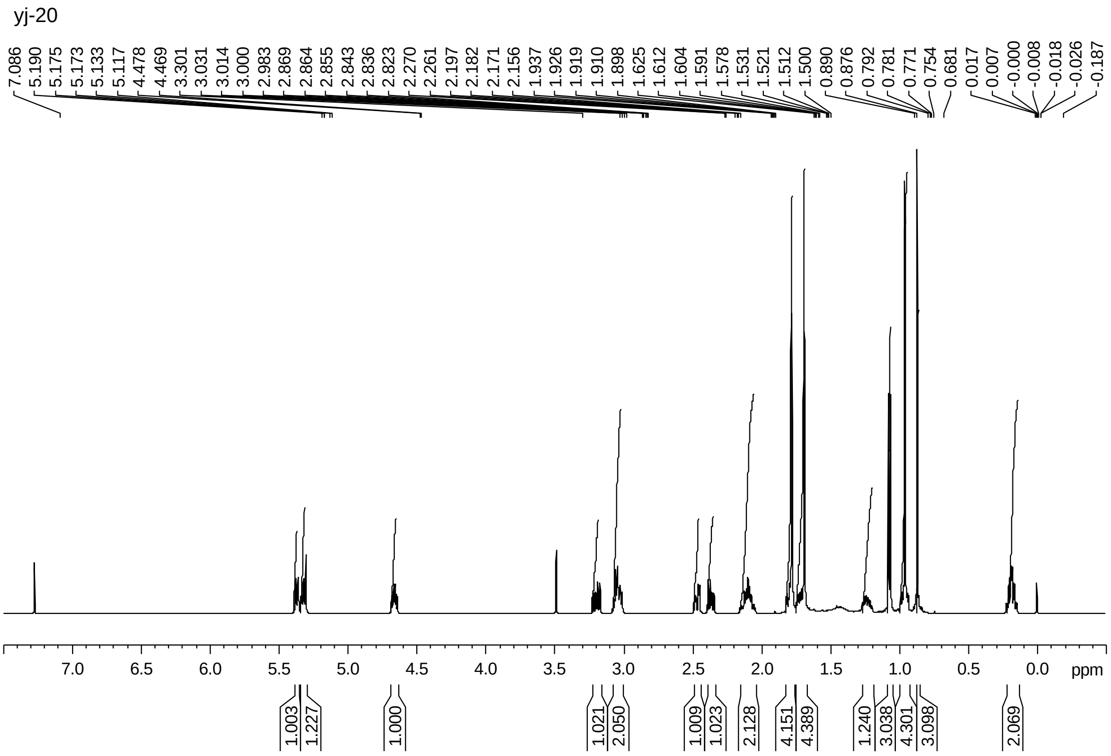

Figure S34. ^{13}C NMR spectrum (125 MHz) of compound **5** in $CDCl_3$.

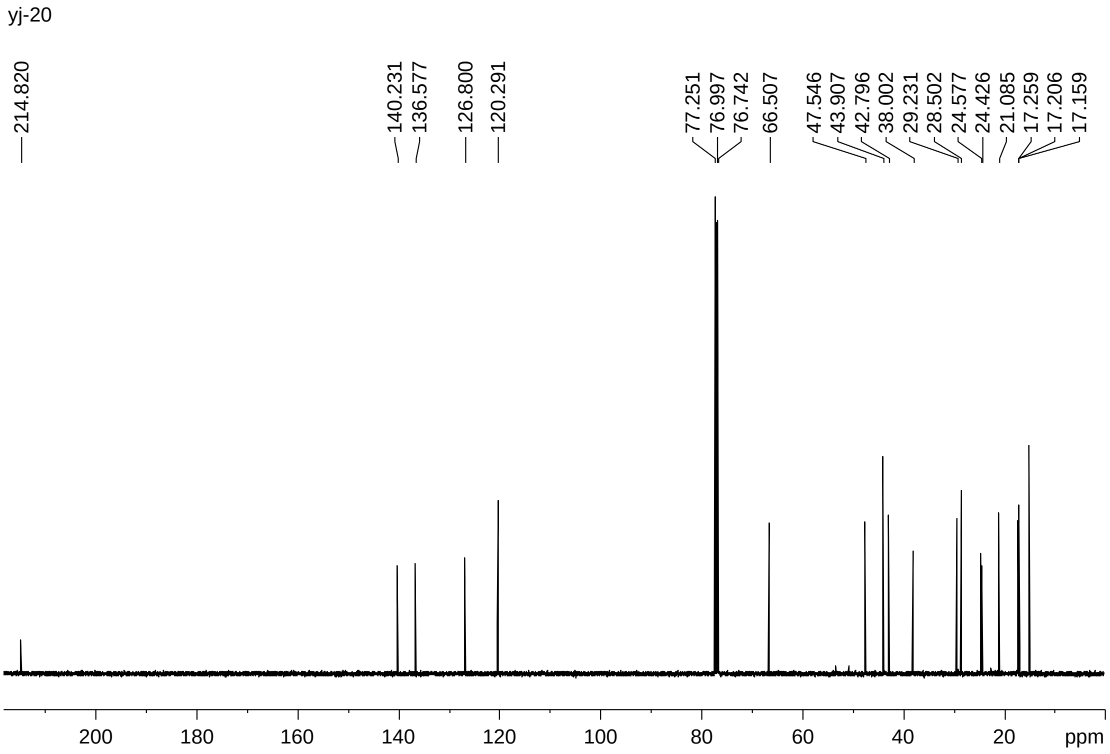

Figure S35. HSQC spectrum (500 MHz) of compound **5** in CDCl3.

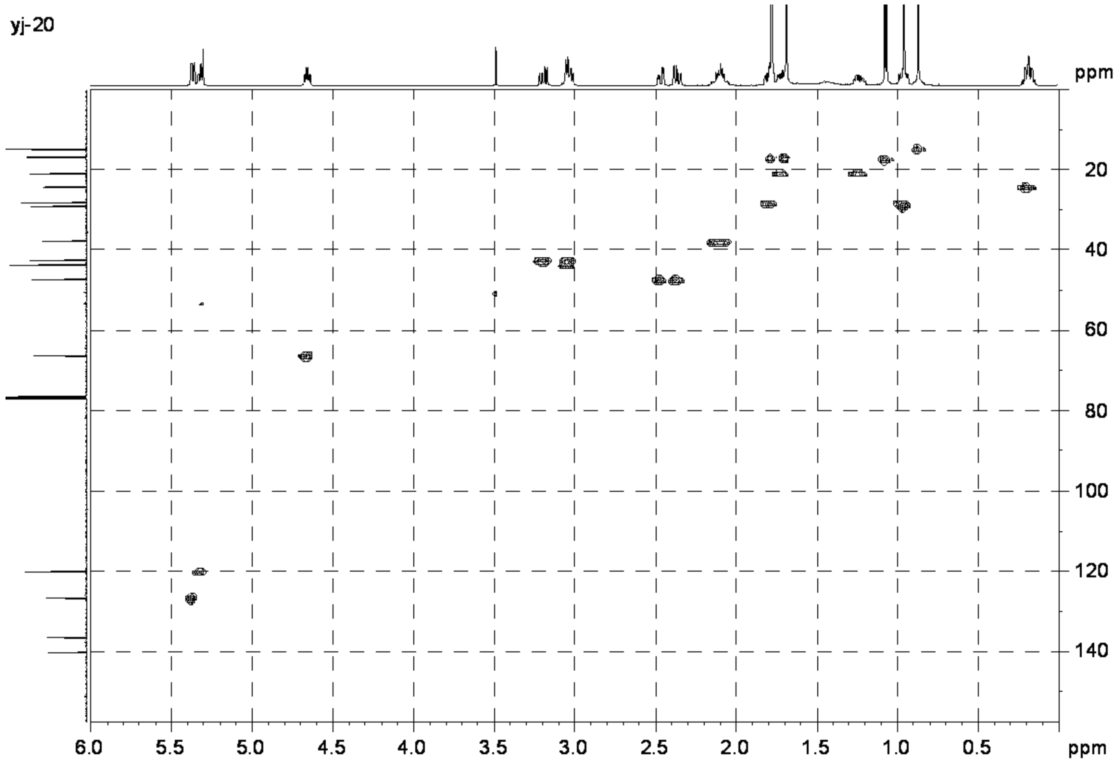

Figure S36. HMBC spectrum (500 MHz) of compound **5** in CDCl3.

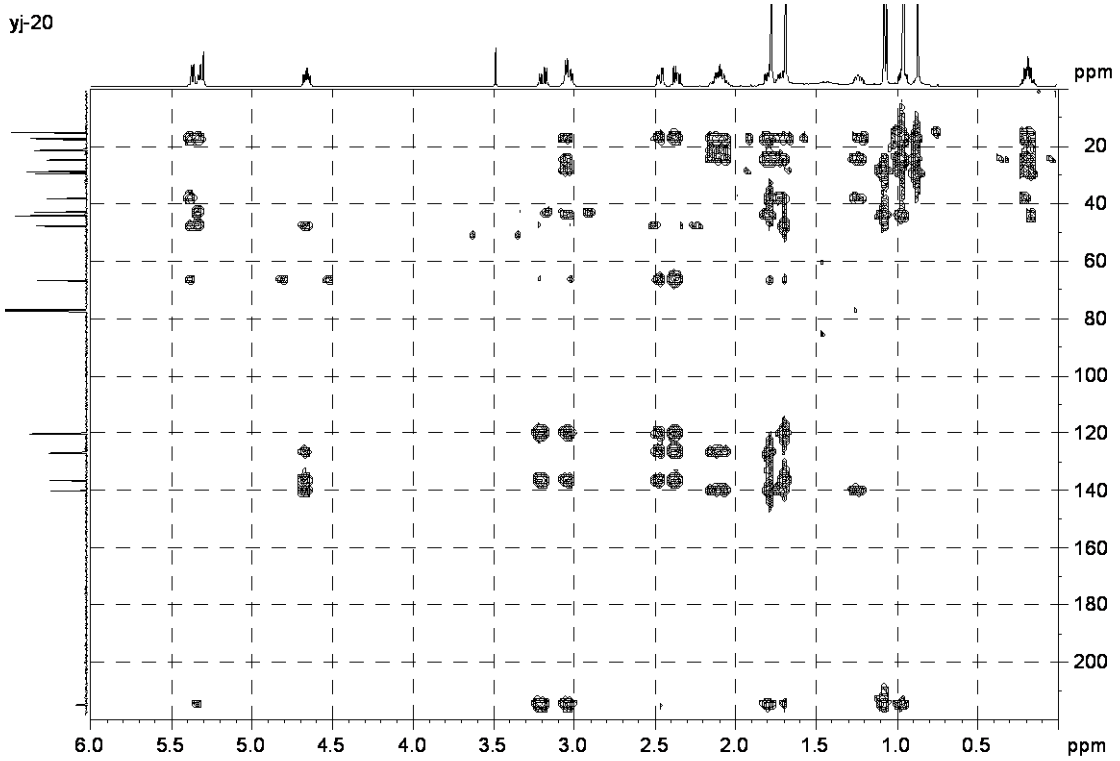

Figure S37. COSY spectrum (500 MHz) of compound **5** in $CDCl_3$.

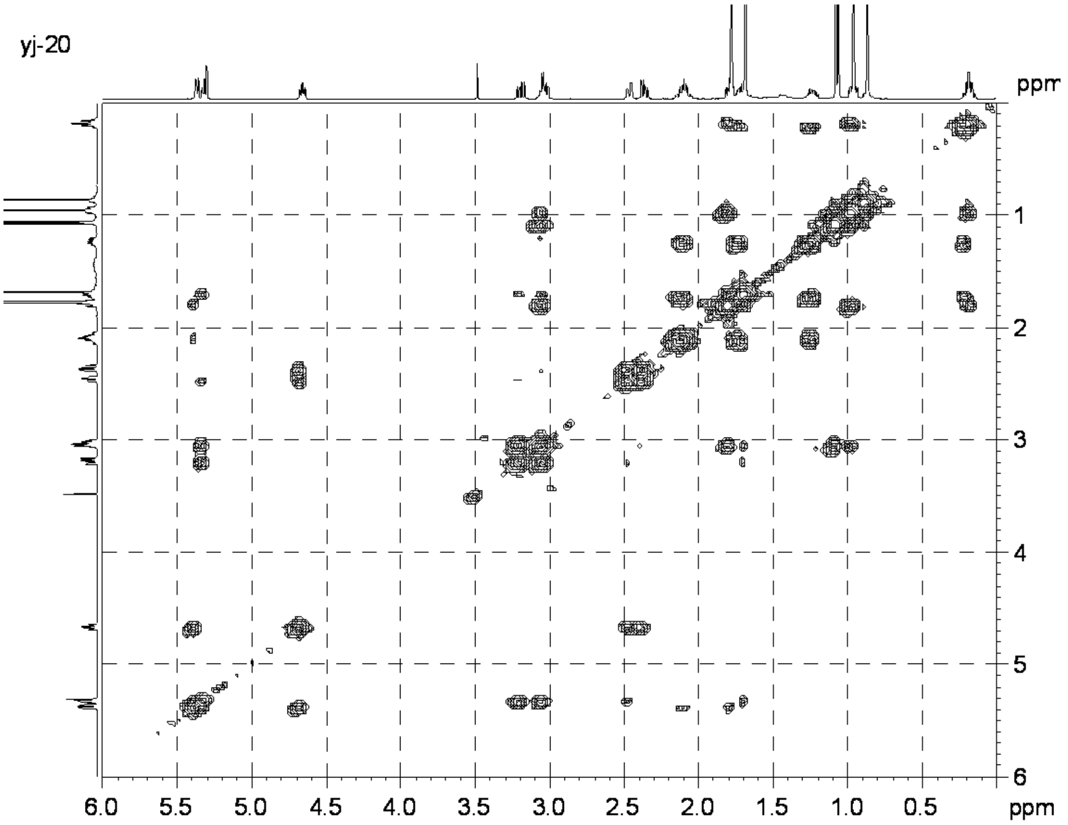

Figure S38. NOESY spectrum (500 MHz) of compound **5** in $CDCl_3$.

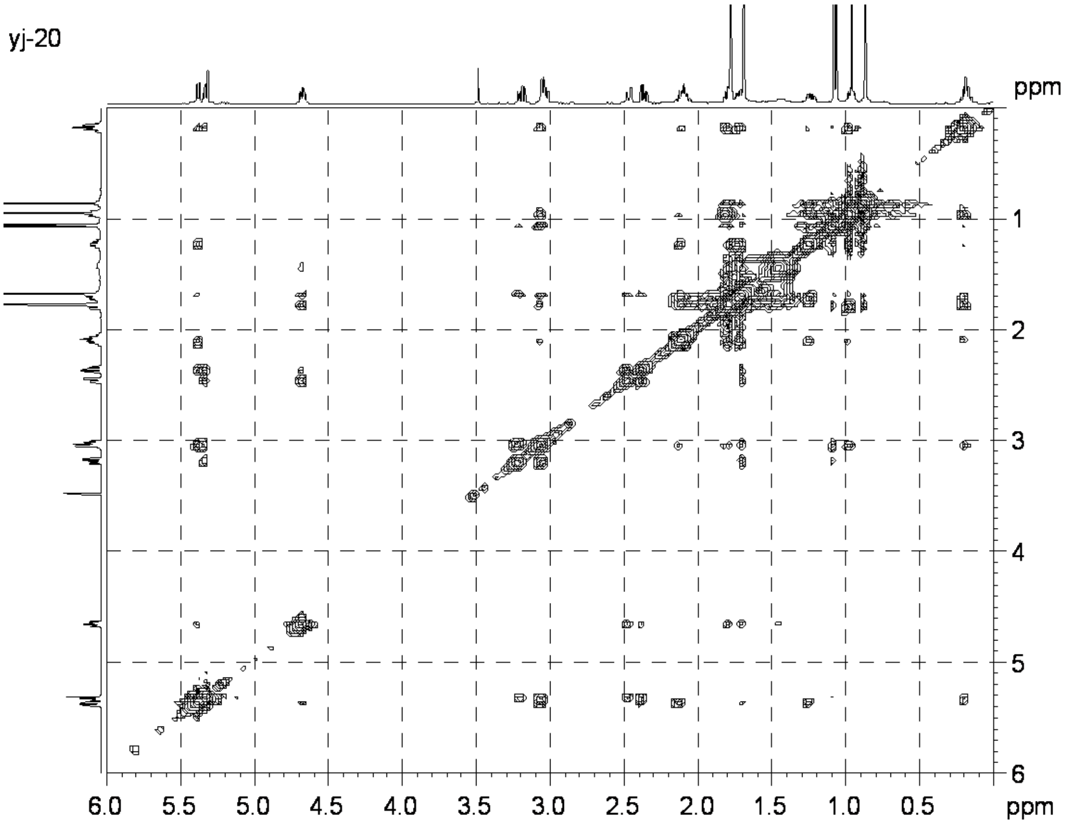

Figure S39. IR spectrum of compound **5**.

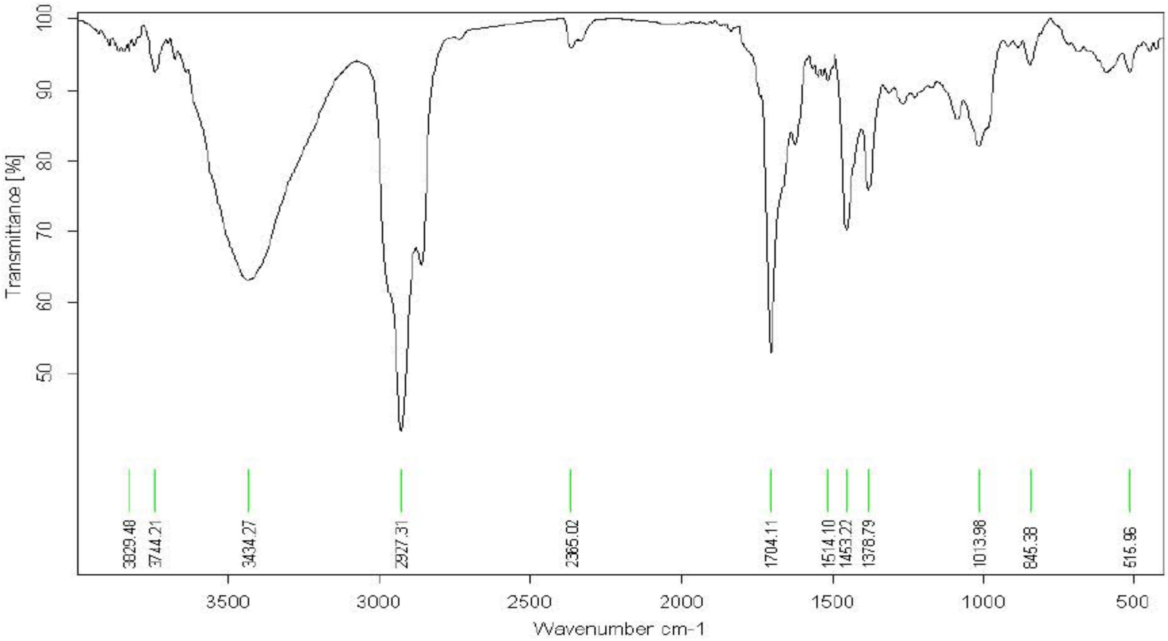

Figure S40. HR-ESIMS spectrum of compound **5**.

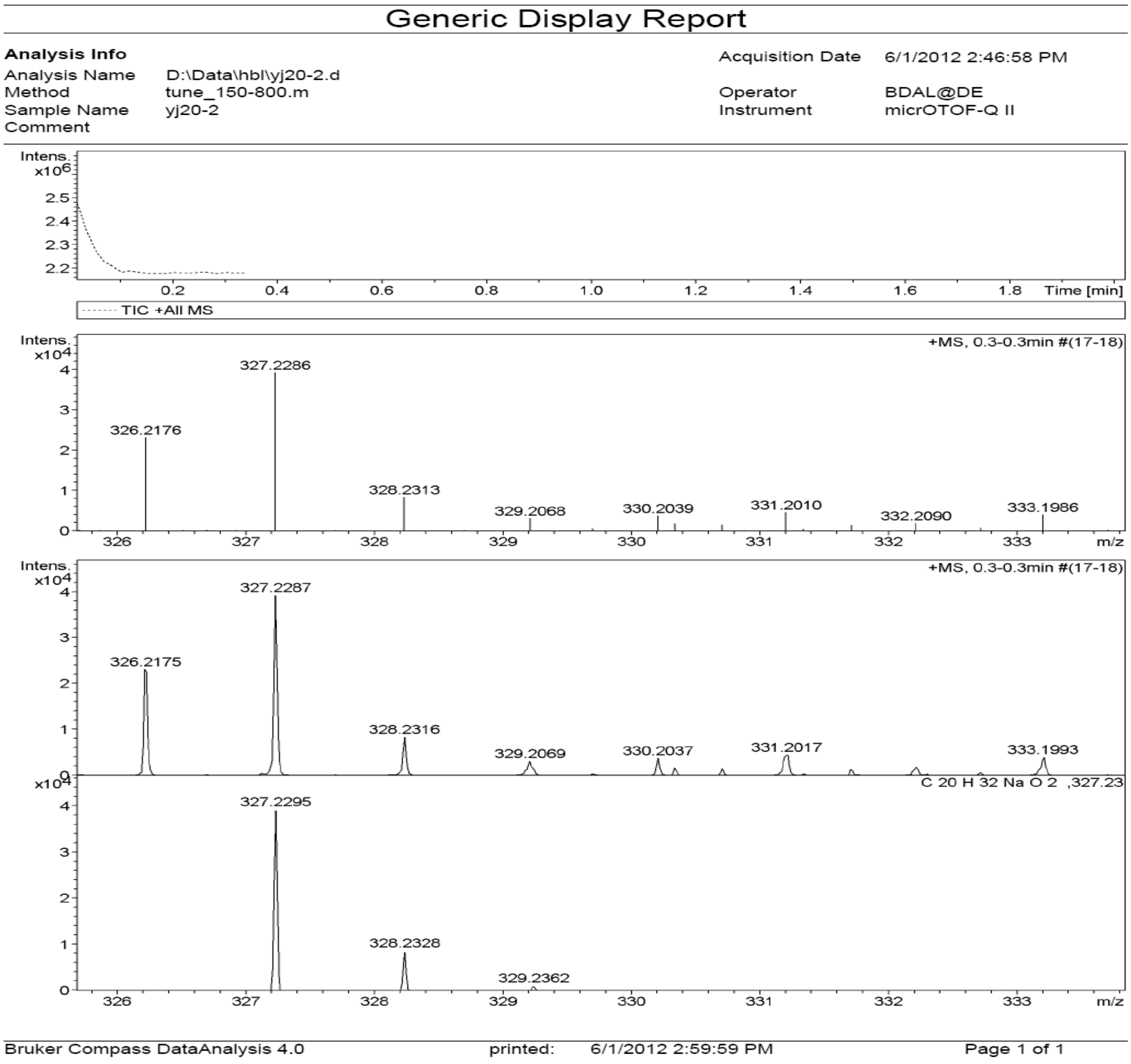

Figure S41. ^{1}H NMR spectrum (500 MHz) of compound **6** in $CDCl_3$.

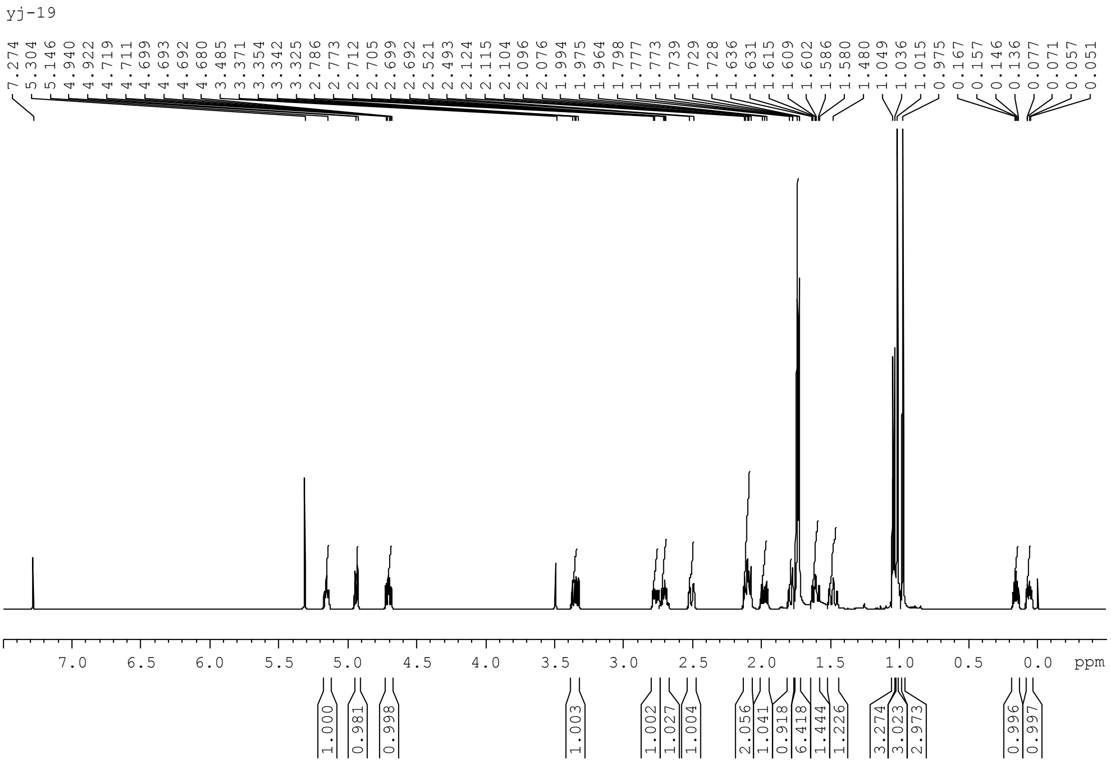

Figure S42. ^{13}C NMR spectrum (125 MHz) of compound **6** in $CDCl_3$.

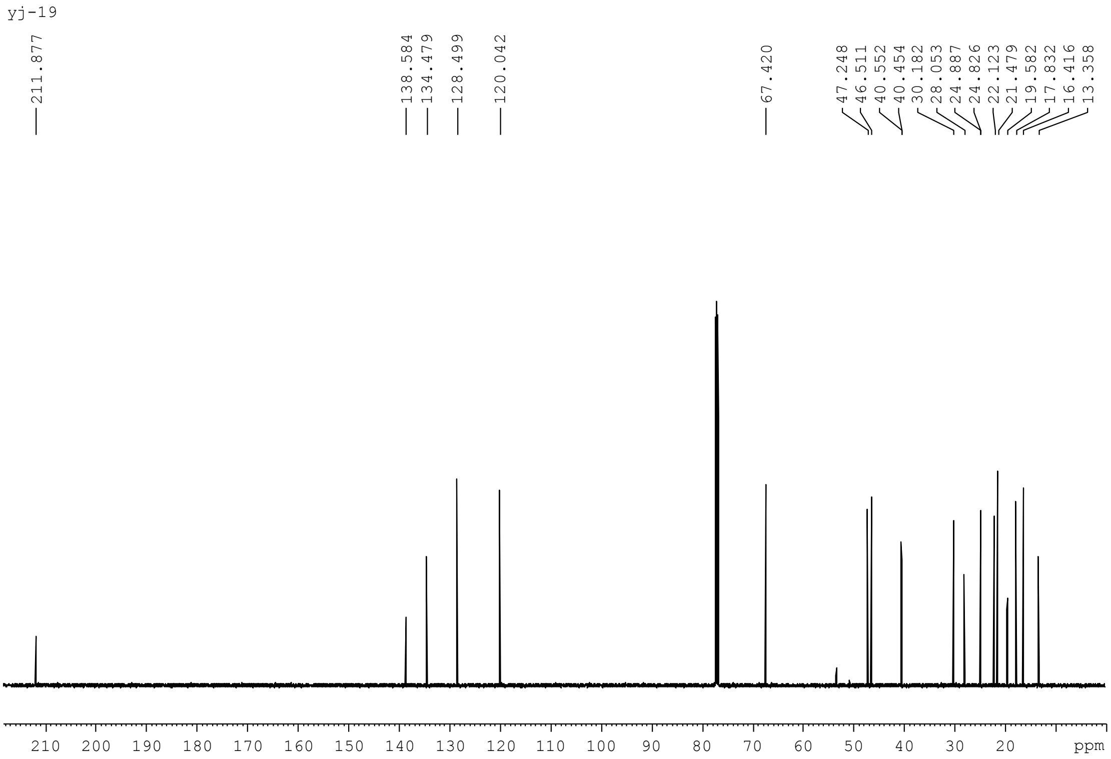

Figure S43. HSQC spectrum (500 MHz) of compound **6** in $CDCl_3$.

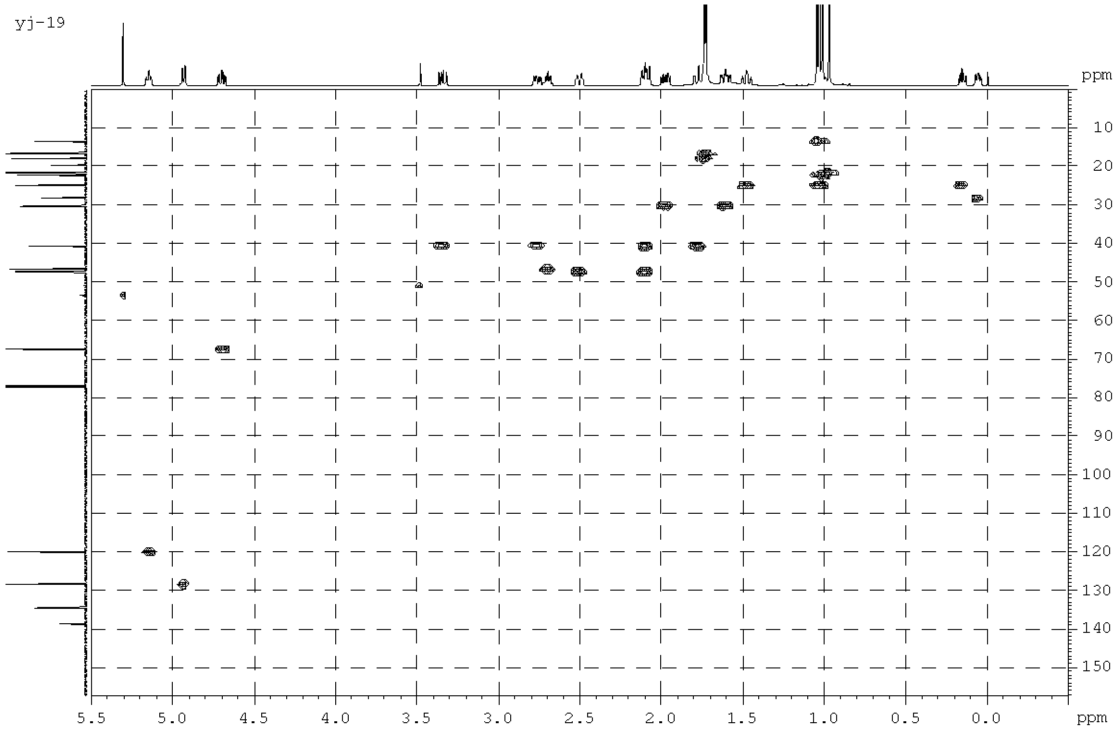

Figure S44. HMBC spectrum (500 MHz) of compound **6** in $CDCl_3$.

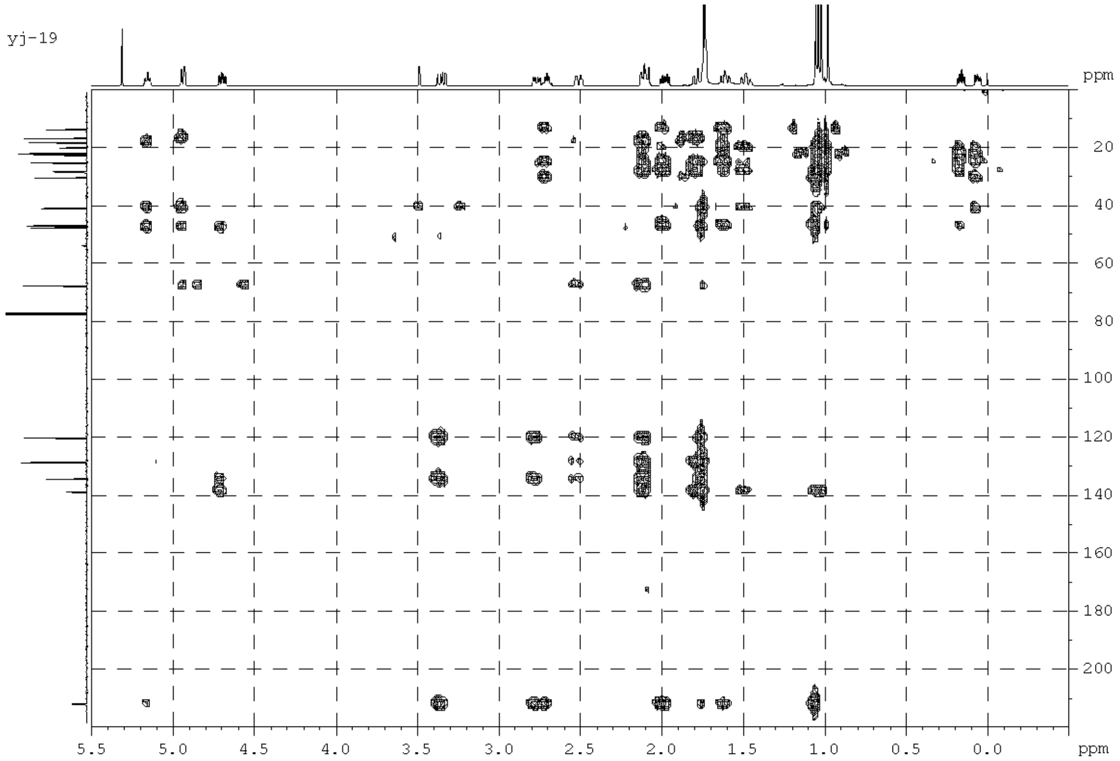

Figure S45. COSY spectrum (500 MHz) of compound **6** in $CDCl_3$.

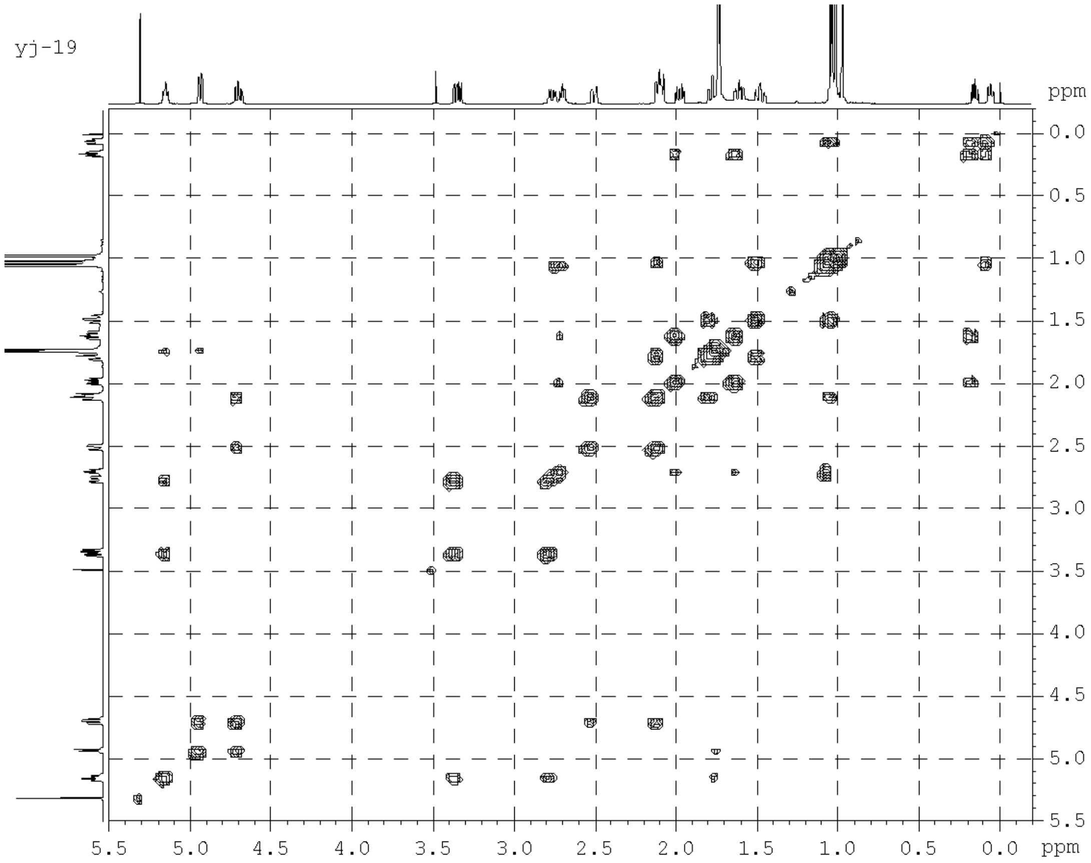

Figure S46. NOESY spectrum (500 MHz) of compound **6** in $CDCl_3$.

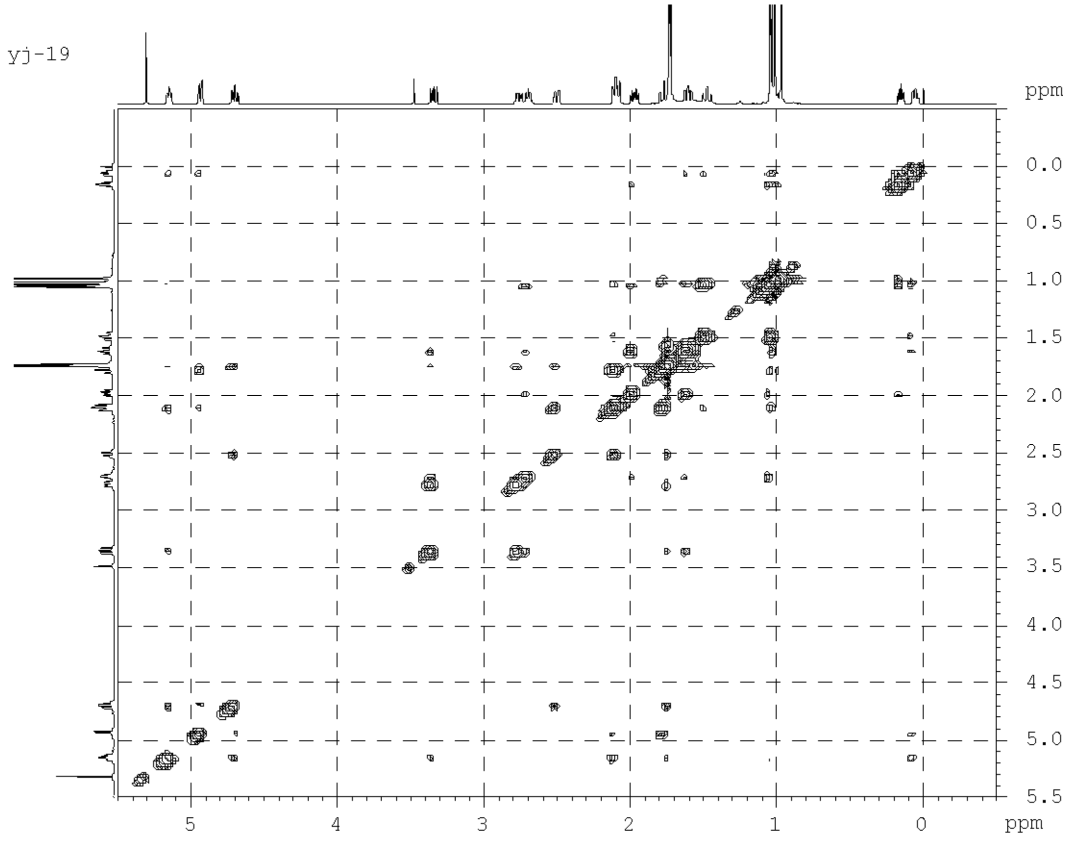

Figure S47. IR spectrum of compound **6**.

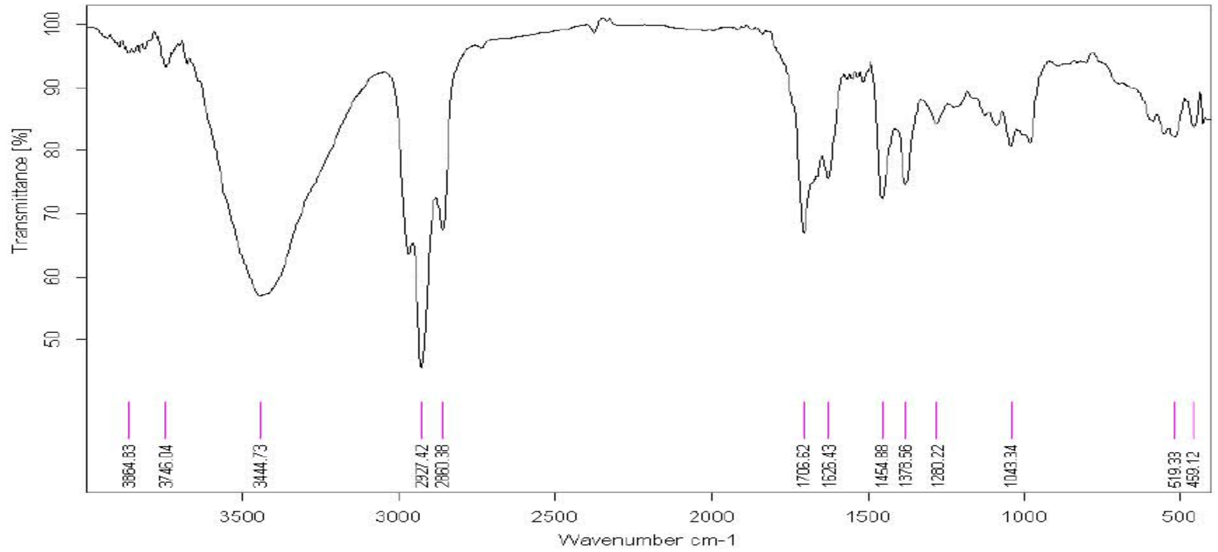

Figure S48. HR-ESIMS spectrum of compound **6**.

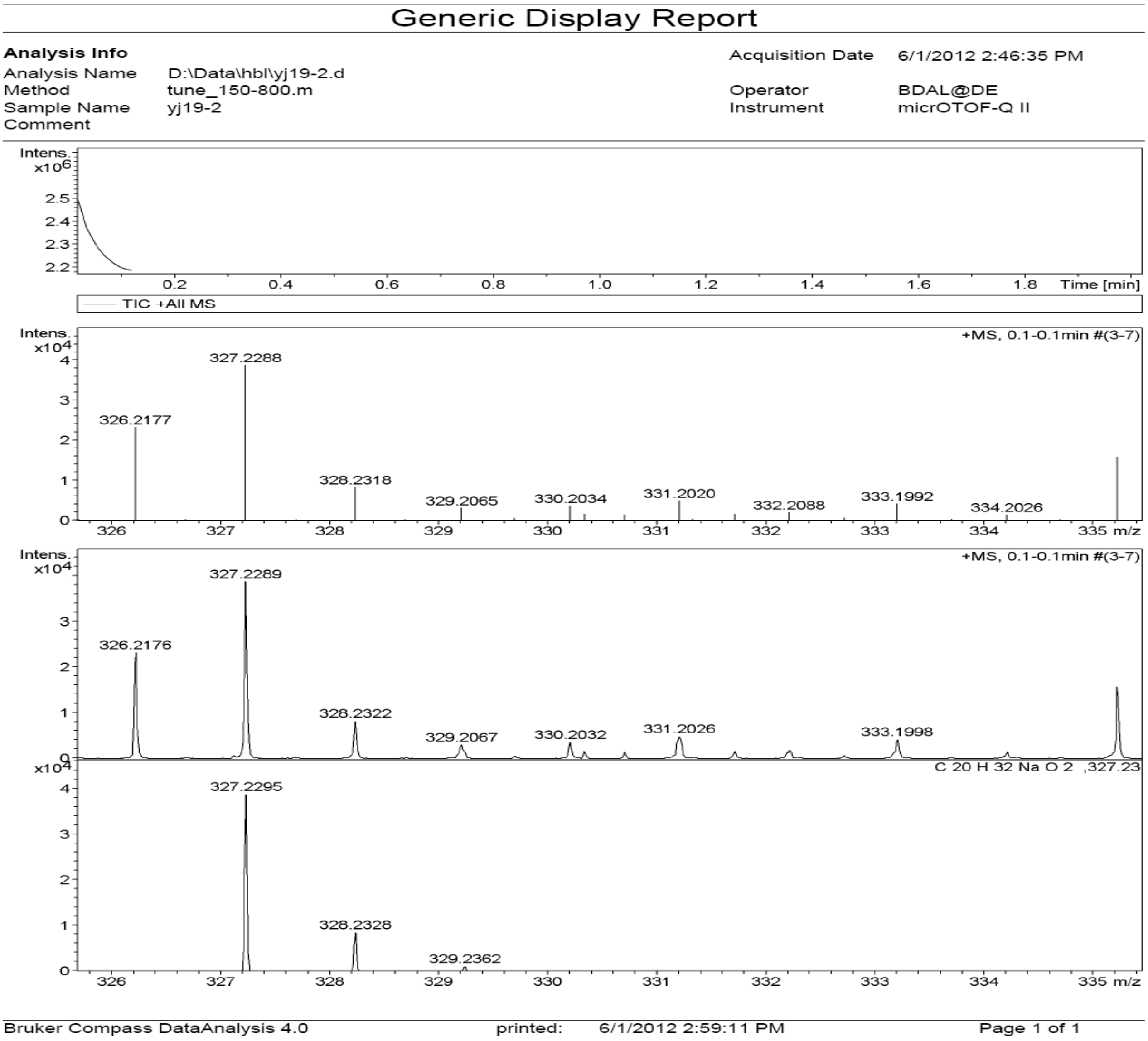

Supplement: Supplementary File 1 — Supplementary Information (PDF, 1305 KB) [file marinedrugs-11-00455-s001.pdf]
